# Influence of TyG Index on Large Vascular Occlusive Stroke Following Endovascular Treatment

**DOI:** 10.1111/cns.70143

**Published:** 2024-12-08

**Authors:** Wei Sun, Huixin Shen, Xiao Wu, Aini He, Xuefan Yao, Fei Chen, Haiqing Song, Xiaoqin Huang

**Affiliations:** ^1^ Department of Neurology, Xuanwu Hospital Capital Medical University Beijing China

**Keywords:** acute ischemic stroke, clinical outcomes, endovascular treatment, triglyceride‐glucose index

## Abstract

**Aims:**

This study aimed to investigate the impact of the triglyceride‐glucose index (TyG index) on clinical consequences in individuals with large vascular occlusion (LVO)‐induced acute ischemic stroke (AIS) following endovascular treatment (EVT).

**Methods:**

We conducted a single‐center retrospective cohort study, including AIS with LVO who underwent EVT. Patients were categorized into TyG index groups, calculated as “(fasting triglyceride [mg/dL] × fasting blood glucose [mg/dL]/2).” Clinical outcomes were assessed, including poor outcome (modified Rankin Scale [mRS] > 2 [3–6]) at 90 days, early neurological deterioration (END), symptomatic intracranial hemorrhage (sICH), and 90‐day mortality after EVT. Logistic regression and restricted cubic splines (RCS) were used to examine the relationship between the TyG index and clinical outcomes. Receiver operating characteristic (ROC) curve was constructed to evaluate the prognostic capacity of the TyG index.

**Results:**

A total of 424 patients were included. Higher TyG levels were associated with worse functional outcome at 90 days (per unit: *p* = 0.006), sICH (per unit: *p* = 0.002, T3 versus T1: *p* = 0.004), and 90‐day mortality (T2 versus T1: *p* = 0.011, T3 versus T1: *p* = 0.029) in logistic regression. A RCS model revealed a linear association between the TyG index and poor outcome at 90 days, sICH, and 90‐day mortality (*p* for nonlinearity > 0.05). In ROC curve analysis, the traditional risk factors model (area under the curve [AUC]: 0.824, 95% CI: 0.784–0.859) was outperformed by the conventional risk factors + TyG index model (AUC: 0.845, 95% CI: 0.807–0.878) in predicting poor outcome (*p* = 0.021).

**Conclusion:**

A higher TyG index is associated with worse clinical outcomes in LVO‐induced AIS patients after EVT. Additionally, the TyG index enhances risk prediction of traditional risk factors for poor outcome.

## Graphical abstract

This single‐center retrospective cohort study indicates that the triglyceride‐glucose index (TyG index), as a marker of insulin resistance, was significantly linked to worse functional outcome, increased mortality at 90 days, and a higher risk of symptomatic intracranial hemorrhage (sICH) in patients with acute ischemic stroke postendovascular treatment. In addition, the TyG index was not significantly associated with early neurological deterioration (END).

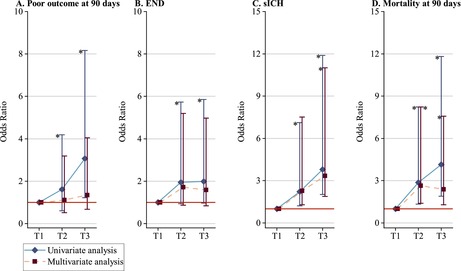

## 1 Introduction

Stroke ranks among the leading global causes of disability and death, with ischemic strokes accounting for approximately 85% of cases [1, 2]. Endovascular treatment (EVT) stands as the recommended standard of care for acute ischemic stroke (AIS) involving large vascular occlusion (LVO), offering swift restoration of blood flow [3, 4]. Recent studies have demonstrated significant clinical advantages associated with early EVT for AIS patients [5, 6, 7, 8]. Nonetheless, the incidence of adverse clinical outcomes remains relatively high [9]. Insulin resistance (IR) is a recognized pivotal risk factor for cardiovascular and cerebrovascular diseases [10, 11, 12, 13]. The triglyceride‐glucose index (TyG index) has emerged as a consistent and accessible surrogate marker for IR in standard clinical practice [14, 15, 16]. Recent investigations have linked the TyG index to poor outcomes following reperfusion therapies in AIS patients [17]. However, it remains unclear that the relationship between the TyG index and clinical outcomes in AIS patients who undergo EVT. Therefore, this study aimed to investigate the impact of the TyG index on clinical outcomes in AIS patients with LVO following EVT.

## 2 Methods

### 2.1 Study Design and Patient Selection

We conducted a retrospective cohort study at Xuanwu Hospital of Capital Medical University from January 2019 to September 2021, examining patients with LVO‐induced AIS after undergoing EVT. The study was approved by the Ethics Committees of Xuanwu Hospital, and all participants or their legal representatives provided informed consent.

The inclusion criteria defined that participants were ≥ 18 years old, diagnosed with AIS attributed to LVO, admitted within 6 h after symptom onset or within 6–24 h after symptom onset if they met the perfusion mismatch, and received EVT following the standard protocol [18, 19].

The exclusion criteria encompassed individuals who underwent angiography alone without subsequent treatment, those without available fasting triglyceride and fasting blood glucose data, those lacking follow‐up brain imaging (computed tomography or magnetic resonance imaging) at 24 h post‐EVT or upon neurological deterioration, and those missing National Institutes of Health Stroke Scale (NIHSS) at 24 h and modified Rankin Scale (mRS) scores at 90 days post‐EVT.

### 2.2 Data Collection and Definition

Baseline data encompassed demographics (age, gender, body mass index [BMI]), vascular risk factors (prestroke, hypertension, diabetes mellitus, hyperlipidemia, coronary artery disease, atrial fibrillation, smoking, and drinking), systolic blood pressure (SBP) and diastolic blood pressure (DBP) upon admission, neurological deficit, Alberta Stroke Program Early Computed Tomography Score (ASPECTS), occlusion site, stroke etiology based on the Trial of Org10172 in Acute Stroke Treatment (TOAST) [20], intravenous thrombolysis (IVT) prior to EVT, tirofiban treatment, reperfusion post‐EVT, early neurological deterioration (END), symptomatic intracranial hemorrhage (sICH), mRS at 90 days post‐EVT, and laboratory tests, including neutrophil to serum glucose level, serum lipid levels, lymphocyte ratio (NLR), and fibrinogen.

Neurological deficit was assessed using the NIHSS, and END was defined as an increase of at least 4 points in the NIHSS score compared with the baseline within 24 h post‐EVT [21, 22]. Patients who presented with the baseline NIHSS scores of < 15 and ≥ 15 were categorized into the mild‐to‐moderate and severe groups, respectively [23, 24]. ASPECTS was evaluated from admission brain noncontrast‐enhanced CT images. The occlusion site was assessed upon admission using brain computed tomographic arteriography (CTA). The decision to employ tirofiban during EVT was at the discretion of the interventionalist, with the dose and duration adjusted based on the patient's condition. Successful reperfusion was defined as a modified Thrombolysis in Cerebral Infarction (mTICI) score of 2b or 3, while unsuccessful reperfusion was defined as an mTICI score of 0‐2a [25]. sICH classification followed the Heidelberg Bleeding Classification, requiring an association of ICH with specific conditions, including: (1) an elevation in NIHSS score by > 4 points relative to the previous level; (2) an elevation in NIHSS score by > 2 points in a single category; (3) decline resulting in intubation, hemicraniectomy, external ventricular drain placement, or other major intervention. The symptom deterioration could not be accounted for by causes other than ICH [26]. Functional outcomes were evaluated by mRS at 90 days, categorizing good and poor outcome as mRS of 0–2 and > 2 (3–6), respectively [27].

### 2.3 Grouping

The TyG index was calculated using the formula: (fasting triglyceride [mg/dL] × fasting blood glucose [mg/dL]/2) [14]. Patients were categorized into three groups based on two TyG index levels: T1 (*n* = 141, 6.79 ≤ TyG index < 8.45), T2 (*n* = 145, 8.45 ≤ TyG index ≤ 8.98), and T3 (*n* = 138, 8.98 < TyG index ≤ 11.04).

### 2.4 Clinical Outcomes

The primary clinical outcome was poor outcome (defined as mRS > 2 [3, 4, 5, 6]) at 90 days post‐EVT. The secondary clinical outcomes were END, sICH, and mortality at 90 days post‐EVT.

### 2.5 Statistical Analysis

Continuous variables were assessed for normality and uniform variance using the Shapiro–Wilk and Levene tests, respectively. For normally distributed and uniformly varying variables (expressed by *x* ± *s*), one‐way analysis of variance was utilized. Non‐normally distributed continuous variables were expressed as median with interquartile range (IQR) and compared using the Kruskal–Wallis nonparametric test. Categorical variables were presented as counts and percentages, with comparisons performed using the Pearson *χ* ^2^ or Fisher test. Pairwise comparisons among multiple groups were conducted using the Bonferroni test.

We used the binary logistic regression analysis to evaluate the effect of TyG index on outcomes, with adjusted odds ratio (OR) and corresponding 95% confidence intervals (CI) reported. Univariate analysis was performed to identified significant factors affecting outcomes, which were subsequently included in multivariate logistic regression approaches. In addition, we performed tests for linear trend for TyG index and outcomes by entering the median value of each category of TyG index as a continuous variable in the models.

Nonlinear relationships were assessed using restricted cubic splines (RCS) with three knots, and the significance of the nonlinear model was determined by likelihood ratio tests. The poor outcome, mortality, sICH, and END were plotted, where OR corresponding to the 95% CI by the TyG index level of 7–11 was predicted with the assistance of the unadjusted and adjusted model. A plot was generated connecting multiple prediction points.

Furthermore, subgroup analysis was conducted to explore whether the association between TyG index and clinical outcomes was modified by diabetes mellitus, NIHSS on admission, and TOAST classification.

Receiver operating characteristic (ROC) curves were constructed to evaluate whether including the TyG index improved the prognostic capacity of models holding typical risks for poor outcome. Additionally, two other metrics, the net reclassification index (NRI) and integrated discrimination improvement (IDI), were computed to evaluate the incremental predictive value of the TyG index.

Statistical analyses were performed using IBM SPSS Statistics (Version 26.0), R (Version 4.3.1), R Studio Desktop (Version 2023.06.1 + 524), and MedCalc (Version 20.1.0), with *p* < 0.05 considered statistically significant.

## 3 Results

### 3.1 Patient Characteristics

**FIGURE 1:**
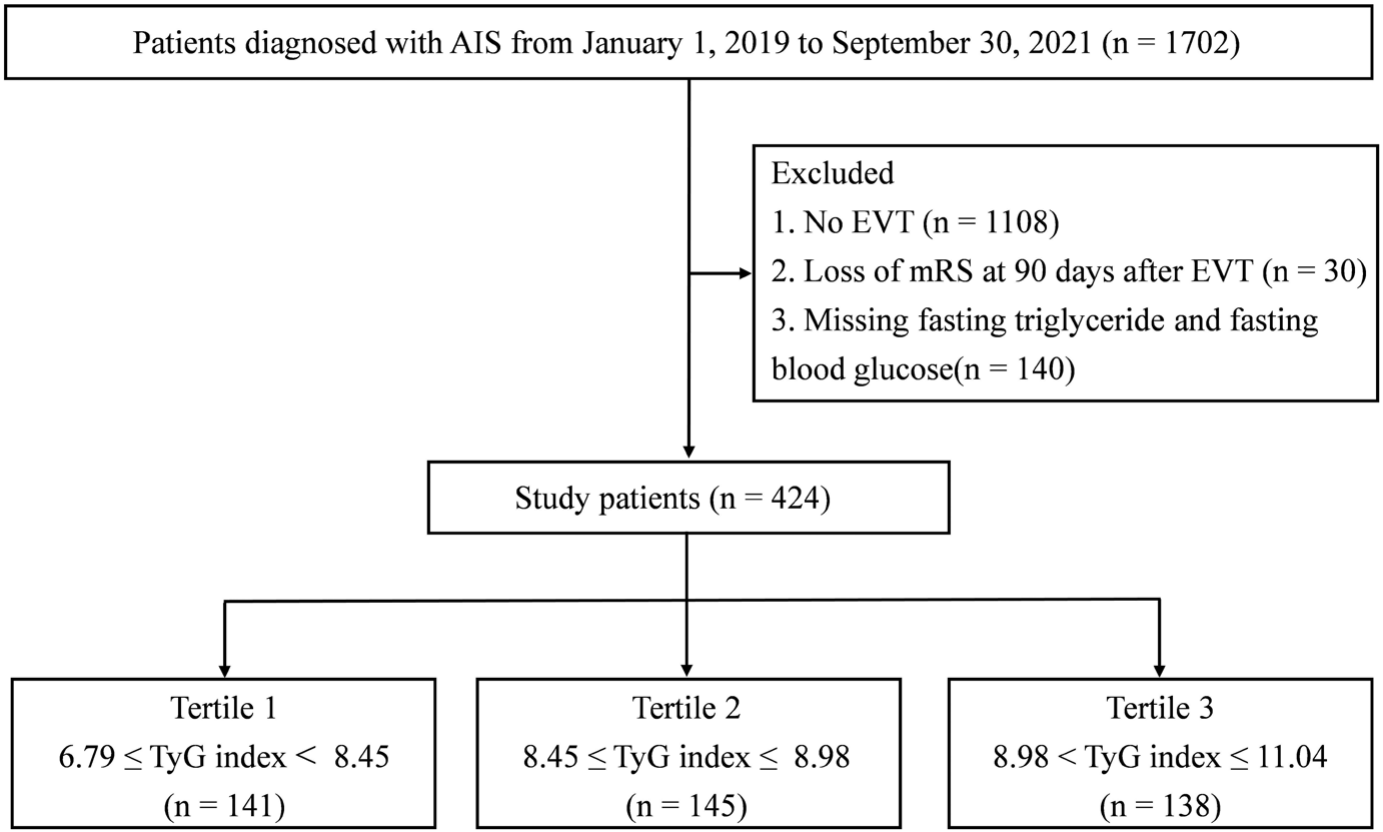
Flowchart of patient selection. AIS, acute ischemic stroke; EVT, endovascular treatment; mRS, modified Rankin Scale; TyG index, triglyceride‐glucose index.

Among 594 consecutive patients with LVO‐induced AIS who underwent EVT, 424 were ultimately included (Figure 1). Several key patient characteristics emerged in our study. The median age was 65 years (from 56 to 72), with 70.28% (298) being male. The median NIHSS score at admission was 16 (from 12 to 20), with a median ASPECTS of 8 (from 7 to 9). A total of 304 patients (71.70%) experienced anterior circulation occlusion. Based upon the TOAST classification, 276 patients (65.09%) were established as having large artery atherosclerosis (LAA), 122 (28.77%) as having cardio‐embolism (CE), and 21 (4.95%) presenting with other stroke subtypes. IVT was administered to 81 patients (19.10%) prior to EVT, and 157 patients (37.03%) received tirofiban treatment. Successful reperfusion was achieved in 374 patients (88.21%).

Notably, the study found that TyG index levels divided patients into three distinct groups (T1, T2, and T3), with significant group differences (*p* < 0.05) in BMI, medical history of prestroke, hypertension, diabetes mellitus, SBP, occlusion site, TOAST classification, tirofiban treatment, blood glucose at admission, fasting blood glucose (FBG), and fasting triglycerides (FTG). Specifically, patients in groups T2 and T3 exhibited higher BMI, a greater prevalence of prestroke conditions, and hypertension, while those in T3 had higher SBP, more anterior circulation infarctions and LAA subtype. Higher TyG index levels also correlated with diabetes mellitus and elevated blood glucose at admission. This research highlights the potential impact of the TyG index on AIS outcomes and prompts questions about its role in treatment and prognosis, as well as its links to other risk factors such as obesity and diabetes.

The characteristics representing the complete set of patients and the three groups are identified in Tables 1 and 2.

**TABLE 1:** Baseline characteristics among groups categorized by the TyG index.

	Total (*n* = 424)	T1 (*n* = 141)	T2 (*n* = 145)	T3 (*n* = 138)	*p* value
Demographics					
Age, years (IQR)	65 (56, 72)	66 (58, 74)	63 (55, 71)	66 (56, 72)	0.072
Gender, male, *n* (%)	298 (70.28)	109 (77.30)	100 (68.97)	49 (64.49)	0.058
BMI, kg/m^2^ (IQR)	25.02 (23.44, 27.68)	24.20 (22.63, 26.69)	25.39 (23.77, 27.76)^a^	25.95 (23.86, 28.08)^b^	< 0.001
Medical history					
Prestroke, *n* (%)	120 (28.30)	37 (26.24)	33 (22.76)	50 (36.23)^b^	0.033
Hypertension, *n* (%)	300 (70.75)	90 (63.83)	98 (67.59)	112 (81.16)^a^, ^b^	0.004
Diabetes mellitus, *n* (%)	119 (28.07)	12 (8.51)	42 (28.97)^a^	65 (47.10)^a^, ^b^	< 0.001
Hyperlipidemia, *n* (%)	167 (39.39)	59 (41.84)	57 (39.31)	51 (36.96)	0.699
Coronary artery disease, *n* (%)	95 (22.41)	25 (17.73)	33 (22.76)	37 (26.81)	0.198
Atrial fibrillation, *n* (%)	98 (23.11)	32 (22.70)	30 (20.69)	36 (26.09)	0.573
Smoking, *n* (%)	177 (41.75)	68 (48.23)	53 (36.55)	56 (40.58)	0.127
Drinking, *n* (%)	145 (34.20)	59 (41.84)	46 (31.72)	40 (28.99)	0.058
NIHSS on admission, (IQR)	16 (12, 20)	15 (11, 19)	16 (12, 20)	16 (12, 22)	0.092
ASPECTS, (IQR)	8 (7, 9)	8 (7, 9)	8 (7, 9)	8 (7, 9)	0.127
Blood pressure on admission, mmHg (IQR)					
SBP	150 (133, 167)	145 (130, 162)	150 (131, 166)	155 (138, 170)^a^, ^b^	0.004
DBP	84 (77, 93)	82 (72, 93)	83 (77, 92)	85 (78, 95)	0.204
Anterior circulation infarction, *n* (%)	304 (71.70)	112 (79.43)	104 (71.72)	88 (63.77)^a^	0.015
Type of TOAST, *n* (%)					
LAA	281 (66.27)	83 (58.87)	91 (62.76)	107 (77.54)^a^, ^b^	0.024
CE	122 (28.77)	48 (34.04)	46 (31.72)	28 (20.29)^a^	
Others	21 (4.95)	10 (7.09)	8 (5.52)	3 (2.17)	
IVT, *n* (%)	81 (19.10)	25 (17.73)	27 (18.62)	29 (21.01)	0.769
Tirofiban treatment, *n* (%)	157 (37.03)	60 (42.55)	40 (27.59)^a^	57 (41.30)^b^	0.014
mTICI 2b ~ 3, *n* (%)	374 (88.21)	125 (88.65)	129 (88.97)	120 (86.96)	0.862

Abbreviations: ASPECTS, Alberta Stroke Program Early Computed Tomography Score; BMI, body mass index; CE, cardio‐embolism; DBP, diastolic blood pressure; IVT, intravenous thrombolysis; LAA, large‐artery atherosclerosis; mRS, modified Rankin Scale; mTICI, modified Thrombolysis in Cerebral Infarction; NIHSS, National Institutes of Health Stroke Scale; SBP, systolic blood pressure; TOAST, Trial of Org10172 in Acute Stroke Treatment.

^a^ Compared with T1, *p* < 0.05.

^b^ Compared with T2, *p* < 0.05.

**TABLE 2:** Laboratory examination among groups categorized by the TyG index.

	Total (*n* = 424)	T1 (*n* = 141)	T2 (*n* = 145)	T3 (*n* = 138)	*p* value
Blood glucose on admission, mmol/L (IQR)	7.20 (6.20, 9.18)	6.60 (5.80, 7.80)	6.90 (6.09, 8.78)^a^	8.65 (6.98, 11.65)^a^, ^b^	< 0.001
FBG, mmol/L (IQR)	7.11 (5.73, 9.27)	5.90 (4.89, 7.07)	6.96 (5.91, 8.41)^a^	9.87 (7.34, 13.89)^a^, ^b^	< 0.001
FTG, mmol/L (IQR)	1.02 (0.75, 1.47)	0.67 (0.54, 0.80)	1.05 (0.91, 1.23)^a^	1.69 (1.31, 2.06)^a^, ^b^	< 0.001
TyG index, (IQR)	8.68 (8.29, 9.18)	8.11 (7.90, 8.29)	8.69 (8.55, 8.83)^a^	9.44 (9.19, 9.78)^a^, ^b^	< 0.001
NLR, (IQR)	6.03 (3.70, 9.87)	5.49 (3.70, 8.45)	6.51 (3.51, 10.21)	6.09 (3.77, 10.19)	0.360
Fibrinogen, g/L (IQR)	3.20 (2.69, 3.73)	3.21 (2.66, 3.73)	3.13 (2.67, 3.81)	3.21 (2.74, 3.76)	0.721

Abbreviations: FBG, fasting blood glucose; FTG, fasting triglycerides; NLR, neutrophil‐to‐lymphocyte ratio; TyG index, triglyceride‐glucose index.

^a^ Compared with T1, *p* < 0.05.

^b^ Compared with T2, *p* < 0.05.

### 3.2 The TyG Index and Outcomes

**FIGURE 2:**
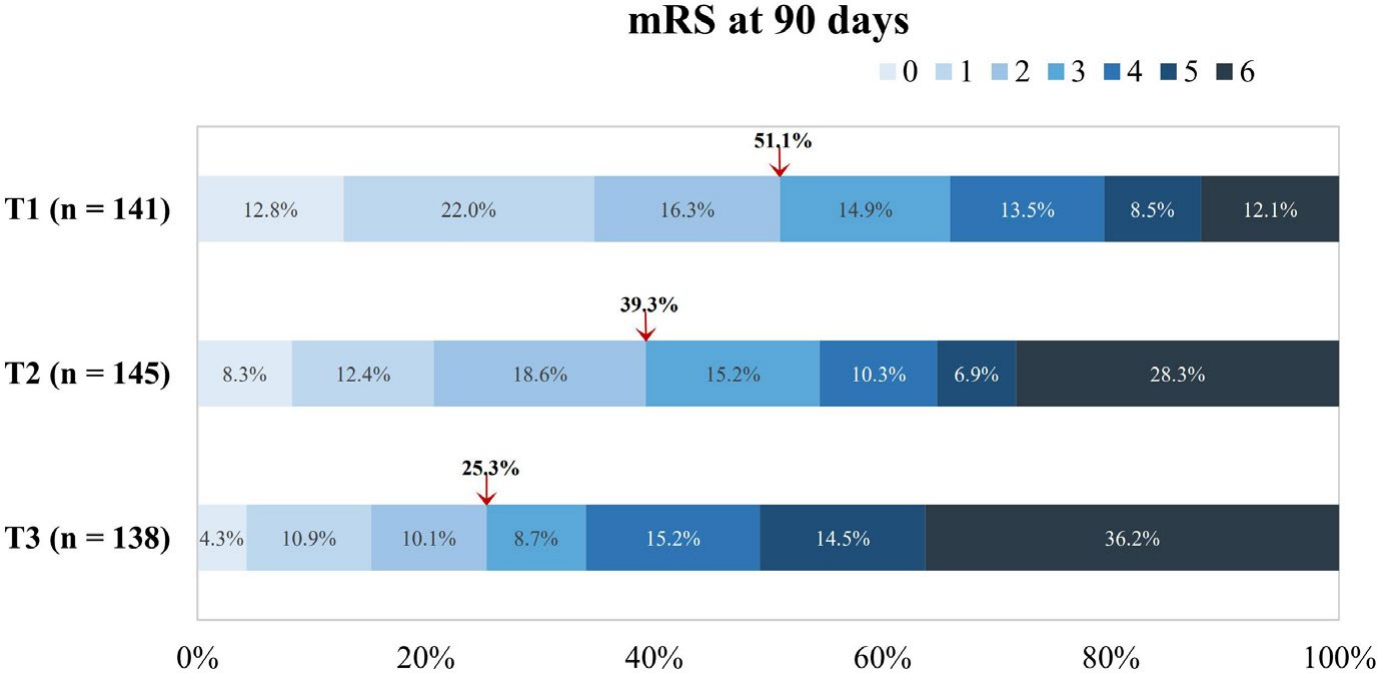
Proportion distribution of different mRS scores at 90 days among groups categorized by the TyG index. There was a statistically significant difference among three groups in the overall distribution of mRS scores (*p* < 0.05). mRS, modified Rankin Scale; TyG index, triglyceride‐glucose index.

Out of the 424 patients, 61.32% experienced unfavorable outcome at 90 days, with substantial differences between the TyG index groups (T1: 48.94%, T2: 60.69%, T3: 74.64%). While there were no notable differences in the occurrence of END, the incidence of sICH varied significantly among the groups (T1: 7.09%, T2: 14.48%, T3: 22.46%), with a similar pattern for mortality at 90 days (T1: 12.06%, T2: 28.28%, T3: 36.23%) (Table 3, Figure 2).

**TABLE 3:** Clinical outcomes among groups categorized by the TyG index.

	Total (*n* = 424)	T1 (*n* = 141)	T2 (*n* = 145)	T3 (*n* = 138)	*p* value
mRS at 90 days, (IQR)	3 (2, 6)	2 (1, 4)	3 (2, 6)^a^	5 (3, 6)^a^, ^b^	< 0.001
Poor outcome at 90 days, *n* (%)	260 (61.32)	69 (48.94)	88 (60.69)	103 (74.64)^a^, ^b^	< 0.001
END, *n* (%)	73 (17.22)	16 (11.35)	29 (20.00)	28 (20.29)	0.078
sICH, *n* (%)	62 (13.62)	10 (7.09)	21 (14.48)	31 (22.46)^a^	0.001
Mortality at 90 days, *n* (%)	108 (25.47)	17 (12.06)	41 (28.28)^a^	50 (36.23)^a^	< 0.001

Abbreviations: END, early neurological deterioration; mRS, modified Rankin Scale; sICH, symptomatic intracranial hemorrhage; TyG index, triglyceride‐glucose index.

^a^ Compared with T1, *p* < 0.05.

^b^ Compared with T2, *p* < 0.05.

**FIGURE 3:**
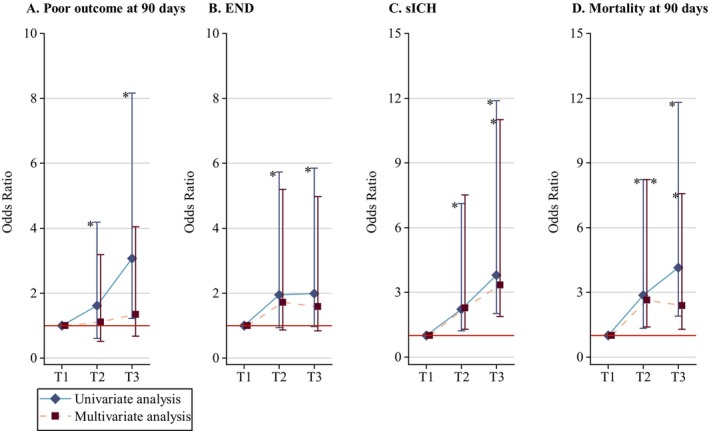
Clinical outcomses measures by tertiles of TyG index. (A) Outcomes measures for poor outcome at 90 days by tertiles of TyG index. (B) Outcomes measures for END by tertiles of TyG index. (C) Outcomes measures for sICH by tertiles of TyG index. (D) Outcomes measures for mortality at 90 days by tertiles of TyG index. *Statistical significance (*p* < 0.05). Adjusted for significant covariates in Tables S1–S4. END, early neurological deterioration; sICH, symptomatic intracranial hemorrhage; TyG index, triglyceride‐glucose index.

Logistic regression analyses, both univariate and multivariate, unveiled that higher TyG levels were linked to worse functional outcome, higher sICH risk, and increased mortality. In the univariate analysis, patients with higher TyG levels experienced worse clinical outcomes than those with lower TyG levels. After adjusting potential influences of age, gender, BMI, medical history of prestroke, hypertension, diabetes mellitus, coronary artery disease, drinking, NIHSS on admission, ASPECTS, SBP, blood glucose on admission, NLR, fibrinogen, anterior circulation infarction, mTICI, END, and sICH (Table S1), higher TyG levels remained associated with higher risk of poor outcome at 90 days (per unit: OR = 1.931, 95% CI = 1.204–3.097, *p* = 0.006). Patients with higher TyG levels also have an increased risk of sICH (when comparing T3 to the reference: OR = 3.345, 95% CI = 1.461–7.660, *p* = 0.004, and per unit: OR = 2.081, 95% CI = 1.312–3.299, *p* = 0.002) after adjusting influencing factors of age, gender, medical history of prestroke, diabetes mellitus, drinking, SBP, and tirofiban treatment (Table S3). Similar results were obtained for mortality at 90 days with significant differences when comparing T2 to T1 (OR = 2.637, 95% CI = 1.243–5.591, *p* = 0.011) and when comparing T3 to T1 (OR = 2.384, 95% CI = 1.093–5.199, *p* = 0.029) after adjustment of age, medical history of prestroke, diabetes mellitus, coronary artery disease, smoking, drinking, NIHSS on admission, ASPECTS, SBP, NLR, fibrinogen, mTICI, END, and sICH (Table S4). There were no significant differences in END among the groups in univariate and multivariate analyses after adjustment of medical history of diabetes mellitus, coronary artery disease, NIHSS on admission, blood glucose on admission, anterior circulation infarction, IVT, and mTICI (Table S2). In univariate analysis, the coefficients for the T1, T2, T3 exhibited a significant increasing trend for the outcomes of poor outcome at 90 days, sICH and mortality at 90 days, while a similar trend in END was not statistically significant. In multivariate analysis, the coefficients for T1, T2, T3 also showed a significant increasing trend for sICH, but a significant decreasing trend for mortality at 90 days. There were no significant differences in other two outcomes across the three groups (Tables 4 and 5, Figure 3).

**TABLE 4:** Univariate and multivariate logistic analysis between TyG index and clinical outcomes.

	Univariate analysis		Multivariate analysis	
	OR (95% CI)	*p* value	OR (95% CI)	*p* value
Poor outcome at 90 days^a^				
T1	Reference		Reference	
T2	1.611 (1.008, 2.575)	0.046	1.114 (0.597, 2.080)	0.734
T3	3.071 (1.851, 5.094)	< 0.001	1.339 (0.662, 2.709)	0.417
*p* for trend	2.324 (1.589, 3.400)	< 0.001	1.246 (0.734, 2.116)	0.416
END^b^				
T1	Reference		Reference	
T2	1.953 (1.009, 3.781)	0.047	1.712 (0.842, 3.483)	0.138
T3	1.989 (1.022, 3.869)	0.043	1.589 (0.746, 3.385)	0.230
*p* for trend	1.569 (0.983, 2.503)	0.059	1.331 (0.773, 2.293)	0.302
sICH^c^				
T1	Reference		Reference	
T2	2.219 (1.005, 4.898)	0.049	2.276 (0.989, 5.241)	0.053
T3	3.795 (1.780, 8.092)	0.001	3.345 (1.461, 7.660)	0.004
*p* for trend	2.555 (1.521, 4.291)	< 0.001	2.272 (1.283, 4.021)	0.005
Mortality at 90 days^d^				
T1	Reference		Reference	
T2	2.876 (1.543, 5.359)	0.001	2.637 (1.243, 5.591)	0.011
T3	4.144 (2.242, 7.661)	< 0.001	2.384 (1.093, 5.199)	0.029
*p* for trend	2.605 (1.711, 3.964)	< 0.001	1.824 (1.071, 3.107)	0.027

Abbreviations: CI, confidence interval; END, early neurological deterioration; OR, odds ratio; sICH, symptomatic intracranial hemorrhage; TyG index, triglyceride‐glucose index.

^a^ Adjusted for significant covariates in Table S1.

^b^ Adjusted for significant covariates in Table S2.

^c^ Adjusted for significant covariates in Table S3.

^d^ Adjusted for significant covariates in Table S4.

**TABLE 5:** Univariate analysis and multivariate logistic analysis between TyG index (per unit) and clinical outcomes.

	Univariate analysis		Multivariate analysis	
	OR (95% CI)	*p* value	OR (95% CI)	*p* value
Poor outcome at 90 days^a^	2.827 (2.000, 3.996)	< 0.001	1.931 (1.204, 3.097)	0.006
END^b^	1.391 (0.967, 2.002)	0.075	1.237 (0.789, 1.940)	0.355
sICH^c^	2.142 (1.448, 3.169)	< 0.001	2.081 (1.312, 3.299)	0.002
Mortality at 90 days^d^	2.232 (1.596, 3.121)	< 0.001	1.505 (0.965, 2.348)	0.072

Abbreviations: CI, confidence interval; END, early neurological deterioration; OR, odds ratio; sICH, symptomatic intracranial hemorrhage; TyG index, triglyceride‐glucose index.

^a^ Adjusted for significant covariates in Table S1.

^b^ Adjusted for significant covariates in Table S2.

^c^ Adjusted for significant covariates in Table S3.

^d^ Adjusted for significant covariates in Table S4.

**FIGURE 4:**
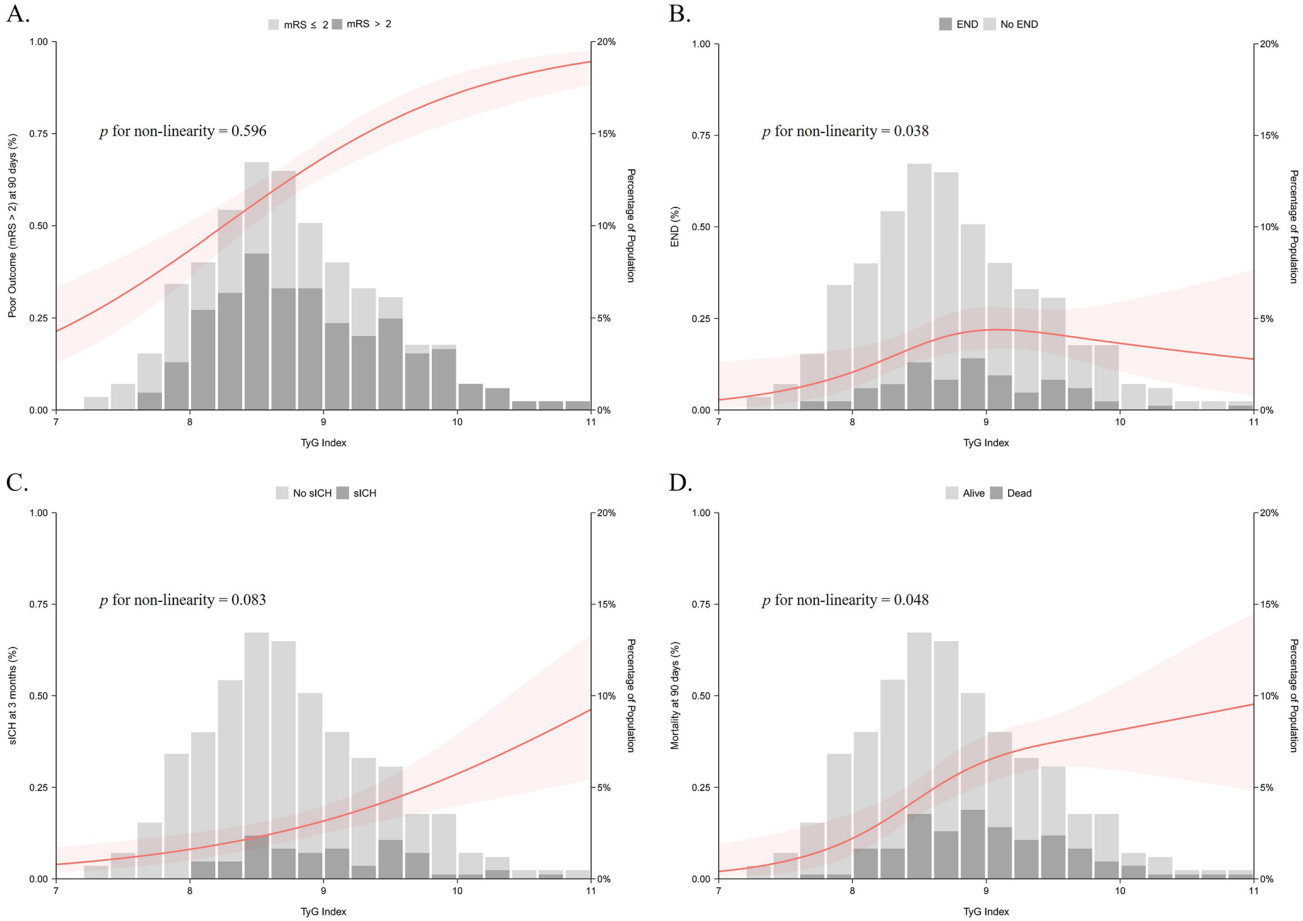
Relationship between the TyG index and clinical outcomes. (A) The linear relationship between the TyG index and poor outcome at 90 days. (B) The linear relationship between the TyG index and END. (C) The linear relationship between the TyG index and sICH. (D) The nonlinear relationship between the TyG index and mortality at 90 days. *p* < 0.05 is considered statistically significant. END, early neurological deterioration; sICH, symptomatic intracranial hemorrhage; TyG index, triglyceride‐glucose index.

The RCS model revealed a linear relationship (*p* for nonlinearity = 0.596) between continuous TyG index and likelihood of poor outcome. Similarly, when fitting an RCS model to evaluate the relationship between the TyG index (per unit) and sICH, a linear association was observed (*p* for nonlinearity = 0.083). The RCS model confirmed a nonlinear relationship (*p* for nonlinearity = 0.048) between TyG index (per unit) and mortality. Notably, for the top 50% of patients, an increase in TyG levels was associated with an elevated risk of mortality (*p* = 0.021). However, this association was not statistically significant in the lower 50% of patients. No significant association was found between the TyG index and END, as indicated by a nonlinear relationship in the RCS model (*p* for nonlinearity = 0.038) (Figure 4). After adjusting covariates identified through univariate analysis, consistent with those used in multivariate logistic regression, the RCS model showed a linear relationship between the TyG index and clinical outcomes. with the *p* for nonlinearity greater than 0.05 (Table 6).

**TABLE 6:** Linear relationship between the TyG index and clinical outcomes after adjustment.

	RCS (TyG index)	RCS (TyG index)‘	*p* for nonlinearity
Poor outcome at 90 days^a^	1.446 (0.591, 3.535)	1.603 (0.455, 5.647)	0.463
END^b^	3.114 (0.906, 10.704)	0.310 (0.076, 1.270)	0.103
sICH^c^	6.263 (1.383, 28.351)	0.283 (0.058, 1.384)	0.119
Mortality at 90 days^d^	4.565 (1.230, 16.950)	0.293 (0.072, 1.204)	0.089

Abbreviations: END, early neurological deterioration; RCS, restricted cubic splines; sICH, symptomatic intracranial hemorrhage; TyG index, triglyceride‐glucose index.

^a^ Adjusted for significant covariates in Table S1.

^b^ Adjusted for significant covariates in Table S2.

^c^ Adjusted for significant covariates in Table S3.

^d^ Adjusted for significant covariates in Table S4.

Further subgroup analysis revealed significant effect of TyG index on the clinical outcomes in specific subgroups. We found that higher TyG levels were associated with worse functional outcome in the patients with diabetes mellitus, NIHSS < 15 and CE (*p* < 0.05). The association between higher TyG levels and increased risk of sICH remained significant in the patients with no diabetes mellitus, NIHSS < 15 and LAA (*p* < 0.05). A similar correlation between higher TyG levels and higher mortality was also found in the subgroup of no diabetes mellitus, NIHSS ≥ 15 and CE (*p* < 0.05). Remarkably, in the subgroup of NIHSS < 15, the TyG levels was significantly associated with END (Tables S6–S9, Figure S1). Both univariate and multivariate RCS model revealed a linear relationship (*p* for nonlinearity > 0.05) between continuous TyG index and likelihood of worse clinical outcomes in all subgroups, exceptions included nonlinear associations with functional outcome in patients with diabetes mellitus, sICH in patients with NIHSS < 15, and END in the CE subgroup (*p* for nonlinearity < 0.05) (Figure S2, Table S10).

### 3.3 Incremental Effect of the TyG Index for Predicting Poor Outcome at 90 Days

**FIGURE 5:**
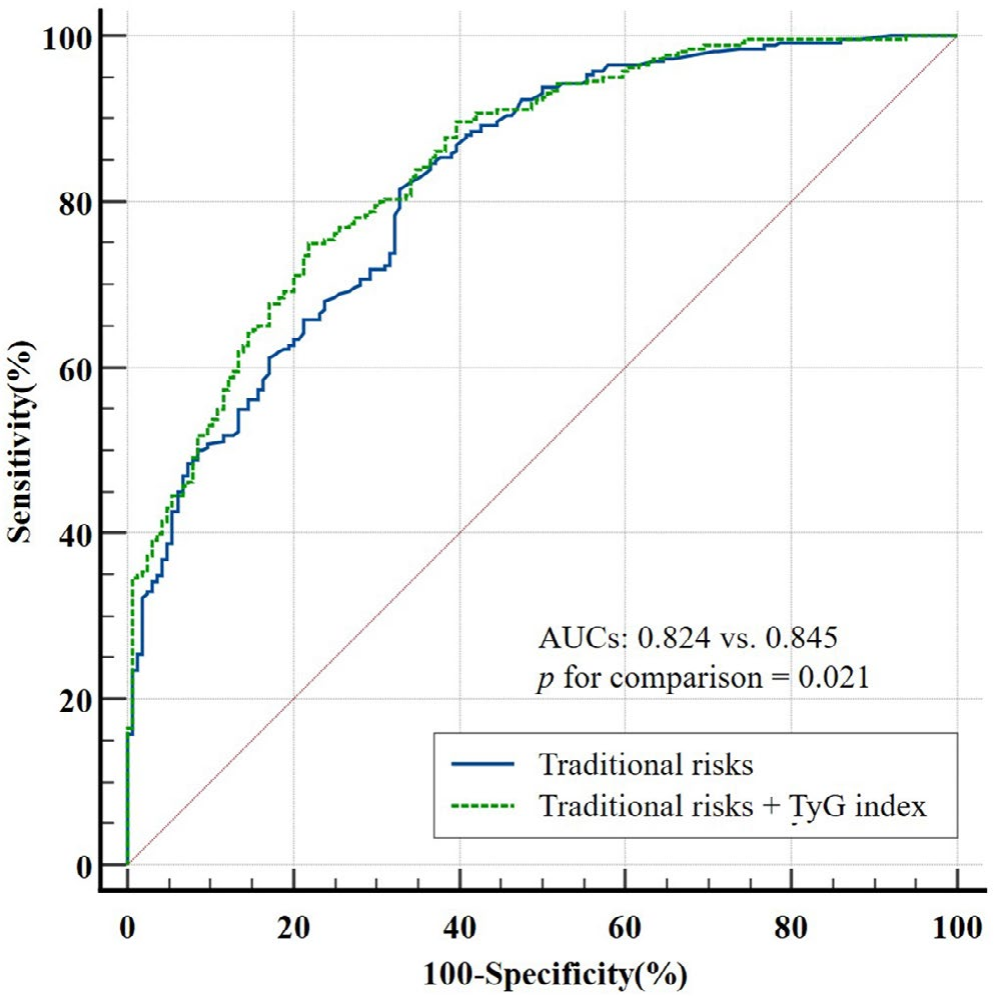
ROC curves for the value of TyG index to predict poor outcome at 90 days. The traditional risk factors model (medical history of prestroke, NIHSS on admission, ASPECTS, mTICI, END, and sICH) was outperformed by the conventional risk factors + TyG index model in predicting poor outcome at 90 days. *p* < 0.05 is considered statistically significant. ASPECTS, Alberta Stroke Program Early Computed Tomography Score; AUC, area under the curve; ROC, receiver operating characteristic; END, early neurological deterioration; mTICI, modified Thrombolysis in Cerebral Infarction; NIHSS, National Institutes of Health Stroke Scale; sICH, symptomatic intracranial hemorrhage; TyG index, triglyceride‐glucose index.

Notably, the ROC curve analysis indicated that the traditional risk factor model's (medical history of prestroke, NIHSS on admission, ASPECTS, mTICI, END, and sICH; Table S5) predictability (area under the curve [AUC]: 0.824, 95% CI: 0.784–0.859) significantly improved when including the TyG index (AUC: 0.845, 95% CI: 0.807–0.878), signifying its enhanced prognostic value for poor outcome at 90 days (*p* = 0.021) (Figure 5). We further calculated more sensitive categorical NRI of 0.107 (95% CI = 0.043–0.170, *p* < 0.001) and IDI of 0.040 (95% CI = 0.020–0.059, *p* < 0.001) (Table 7). The results demonstrated the potential of incorporating the TyG index into the traditional model. These findings raise essential questions about the role of the TyG index as a predictive tool and its clinical implications for AIS management and patient outcomes.

**TABLE 7:** NRI and IDI for the incremental effect of the TyG index for predicting poor outcome at 90 days.

	Categorical NRI		IDI	
	Estimate (95% CI)	*p* value	Estimate (95% CI)	*p* value
Traditional risk factors model		Reference		Reference
Traditional risk factors + TyG index model	0.107 (0.043, 0.170)	< 0.001	0.040 (0.020, 0.059)	< 0.001

Abbreviations: CI, confidence interval; IDI, integrated discrimination improvement; NRI, net reclassification index; TyG index, triglyceride‐glucose index.

## 4 Discussion

Our study observed that elevated TyG index levels were significantly linked to adverse clinical outcomes in patients with LVO‐induced AIS treated who underwent EVT. Specifically, higher TyG index levels were associated with worse functional outcome, an increased risk of mortality at 90 days, and a higher risk of sICH. The predictive model, including the TyG index, improved their performance in forecasting poor outcome.

Previous research has established a strong connection between the TyG index and IR, considering TyG as a proxy and readily accessible marker of IR [14, 15, 16]. IR represents a condition where insulin exerts a reduced biological effect than typically observed [28, 29]. This condition can disrupt glucose metabolism, leading to hyperglycemia, which, in turn, triggers oxidative stress and initiates an inflammatory response that can cause cellular damage. Additionally, IR has a notable impact on systemic lipid metabolism, contributing to the progression of dyslipidemia [10, 30].

The TyG index, resulting from FBG and FTG levels, has been identified as an essential indicator of metabolic syndrome, including conditions such as diabetes, obesity, hypertension, and dyslipidemia [10]. Moreover, the TyG index has shown a strong linkage with carotid atherosclerosis [31] and an elevated risk of cardiovascular issues in patients [10, 11]. Interestingly, a substantial correlation has been noted between IR and the risk of cerebrovascular events. A meta‐analysis encompassing 18 studies and 592,635 patients indicated that a higher TyG index was associated with an increased risk of ischemic stroke (IS) in the general population. And IS patients with a higher TyG index faced a greater risk of stroke recurrence and elevated mortality [12]. Hoshino et al. found that increased TyG index were significantly linked to a higher prevalence of ipsilateral extracranial and intracranial atherosclerotic stenosis and were a notable predictor of nonfatal stroke, nonfatal acute coronary syndrome, and vascular death, accompanied by IS, and transient ischemic attack [32]. Meanwhile, the TyG index was identified to be significantly associated with carotid artery plaques in patients with IS [33]. Furthermore, research has shown a positive correlation between the TyG index and the risk of vascular restenosis following revascularization [34]. The Kailuan cohort study uncovered that a high cumulative TyG index was associated with a higher risk of IS [35]. Previous studies have demonstrated that an elevated TyG index was notably connected to a higher risk of LAA stroke with certain diagnostic value in distinguishing LAA from CE [36, 37], and ischemic stroke recurrence within specific subgroups [38, 39]. Furthermore, the TyG index may be a major risk factor for early‐onset stroke among young populations [40]. Additionally, recent research has revealed that an increased TyG index was related to poor functional outcomes and END in patients with AIS treated with IVT [41, 42, 43, 44]. And Minwoo Lee and colleagues discovered that higher TyG index was associated with poor functional outcomes in patients with AIS of the anterior circulation who received reperfusion therapies [17].

Despite extensive research on the TyG index and its connection to various health conditions, its role in patients with AIS post‐EVT, a crucial component of stroke management, remains unclear. What is particularly noteworthy in our study is the observed association between higher TyG index levels and adverse clinical outcomes in patients with LVO‐induced AIS who underwent EVT. While the underlying mechanism remains incompletely understood, several possible explanations exist for these findings. The TyG index comprises two risk factors, one related to lipids and the other to glucose, which reflect IR. IR can lead to an imbalance in glucose metabolism, resulting in hyperglycemia, causing inflammation and oxidative stress. These pathological metabolic disturbances can expedite the demise of neurons in the ischemic penumbra and compromise the integrity of the blood–brain barrier (BBB), increasing the risk of hemorrhagic transformation [45, 46]. Furthermore, IR can lead to nitric oxide (NO) inactivation and excessive reactive oxidative species (ROS) generation. Aberrant NO secretion damages the vascular endothelium, causing vasomotor dysfunction, while ROS contributes to endothelial dysfunction [47, 48]. These endothelial issues may extend to cerebral microcirculation, which can impair the compensatory ability for acute ischemic injury, impairing the compensatory ability for acute ischemic injury, such as the self‐regulatory function of cerebrovasculature, and exacerbating BBB permeability, which raises the risk of intracranial hemorrhage following reperfusion [49]. Additionally, IR plays a significant role in enhancing platelet activation and increasing tissue factor expression linked to platelet adhesion and aggregation [50]. It also contributes to thrombin generation [51], events linked to thrombosis, and may partially account for the negative prognosis associated with higher TyG index levels. Furthermore, cardiovascular studies have established an inverse relationship between IR and collateral density in response to ischemia [52]. Activation of the renin–angiotensin–aldosterone system and calcium overload, both associated with IR, may also contribute to a poor prognosis [10, 53].

Further subgroup analysis revealed exciting insights into the relationship between TyG index levels and clinical outcomes in specific subgroups. This discrepancy in findings may be due to underlying mechanisms and the possibility that certain risk factors mask the relationship between IR and outcomes. While our exploratory research has begun to elucidate how the TyG index may impact functional consequences in AIS patients following EVT, it is essential to exercise caution when interpreting these findings. The scarcity of existing clinical studies on this subject underscores the need for further research to confirm and expand upon these observations.

This study provides evidence of a connection between the TyG index and clinical outcomes in patients with AIS post‐EVT. These findings can potentially advance our comprehension of latent prediction and risk stratification, which, in turn, can aid in identifying regulatory targets to mitigate adverse clinical outcomes. In the context of risk prediction, our study demonstrated that the incorporation of the TyG index into traditional risk factor models significantly improved the accuracy of predicting poor prognosis. Many studies have already underscored the importance of including multiple risk factors in current prediction models. New risk stratification approaches can be instrumental in personalizing prognosis and enabling the prioritization of high‐risk individuals for more intensive clinical endeavors. The relationship between the TyG index and clinical outcomes indicates that modulating the TyG index may be a reasonable approach to preventing adverse clinical outcomes in patients post‐EVT. As a result, our findings suggest that the inclusion of TyG index predictors in models may be a valuable variable for discerning high‐risk AIS patients post‐EVT, thus facilitating clinical decision‐making.

However, several limitations should be acknowledged in this study. Firstly, the results regarding the association between TyG index (as a continuous variable and categorical variable) and poor outcome at 90 days were inconsistent. We attribute this discrepancy to the loss of information caused by categorical variables, which may reduce the analytical efficiency. This necessitates a cautious interpretation of the findings. Secondly, due to database restrictions, we were unable to compare the TyG index with other established IR measuring techniques. Thirdly, the measurements of triglycerides, glucose, and other parameters were only assessed at baseline, and variations over the follow‐up period were not considered, potentially obscuring the valid and long‐term impacts. Lastly, the potential underdiagnosis of diabetes mellitus, due to an undetected hemoglobin A1c, poses another limitation to our study.

## 5 Conclusion

In summary, the TyG index, as a marker of IR, was significantly linked to worse functional outcome, increased mortality at 90 days, and a higher risk of sICH in patients with AIS post‐EVT. Furthermore, adding the TyG index to standard risk factors significantly enhanced the accuracy of risk forecasting for poor outcome. The findings contribute to our understanding of latent prediction and risk stratification in patients with AIS post‐EVT, and they offer insights that can potentially help to identify regulatory targets for preventing adverse clinical outcomes.

## Author Contributions

W.S.: writing original draft. H.S.: writing review and editing. X.W., A.H., X.Y., F.C.: acquisition and analysis of data. X.H.: conception and design of the study, writing review and editing, funding acquisition. H.S.: conception and design of the study, funding acquisition. All authors approved the work.

## Ethics Statement

This study was approved by the Ethics Committees of Xuanwu Hospital and was conducted according to the principles of the Declaration of Helsinki. All participants or their legal representatives signed informed consent forms for data collection.

## Conflicts of Interest

The authors declare no conflicts of interest.

## Supporting information


Data S1.

## Data Availability

The datasets used and/or analyzed during the current study are available from the corresponding authors upon reasonable request.
