# Supplementary material for: Influence of TyG Index on Large Vascular Occlusive Stroke Following Endovascular Treatment
Source: CNS Neurosci Ther. 2024 Dec 8;30(12):e70143. doi: 10.1111/cns.70143 (PMC11625684; doi:10.1111/cns.70143)
Supplement: Supplementary file 1 — Data S1. [file CNS-30-e70143-s001.docx]

**Table S1.** Baseline and clinical characteristics of patients with poor outcome and good outcome at 90 days.

|  | Poor outcome (n=260) | Good outcome (n=164) | *p* Value |
| --- | --- | --- | --- |
| Demographics |  |  |  |
| Age, years (IQR) | 66 (57, 74) | 63 (53, 71) | 0.002 |
| Gender, male, n (%) | 170 (65.38) | 128 (78.05) | 0.005 |
| BMI, kg/m^2^ (IQR) | 25.68 (23.73, 27.78) | 24.48 (22.86, 27.00) | 0.002 |
| Medical history |  |  |  |
| Pre-stroke, n (%) | 91 (35.00) | 29 (17.68) | < 0.001 |
| Hypertension, n (%) | 193 (62.69) | 107 (65.24) | 0.048 |
| Diabetes mellitus, n (%) | 90 (34.62) | 29 (17.68) | < 0.001 |
| Hyperlipidemia, n (%) | 94 (36.15) | 73 (44.51) | 0.086 |
| Coronary artery disease, n (%) | 71 (27.31) | 24 (14.63) | 0.002 |
| Atrial fibrillation, n (%) | 68 (26.15) | 30 (18.29) | 0.061 |
| Smoking, n (%) | 104 (40.00) | 73 (44.51) | 0.359 |
| Drinking, n (%) | 77 (29.62) | 68 (41.46) | 0.012 |
| NIHSS on admission, (IQR) | 17 (13, 22) | 13 (10, 18) | < 0.001 |
| ASPECTS, (IQR) | 8 (7, 8) | 9 (8, 10) | < 0.001 |
| Blood pressure on admission, mmHg (IQR) |  |  |  |
| SBP | 151 (138, 170) | 144 (129, 160) | < 0.001 |
| DBP | 85 (78, 93) | 82 (73, 92) | 0.164 |
| Laboratory examination |  |  |  |
| Blood glucose on admission, mmol/L (IQR) | 7.60 (6.40, 9.80) | 6.75 (6.00, 8.08) | < 0.001 |
| FBG, mmol/L (IQR) | 7.62 (6.43, 11.41) | 6.16 (5.05, 7.33) | < 0.001 |
| FTG, mmol/L (IQR) | 1.04(0.78, 1.58) | 0.99(0.66, 1.35) | 0.028 |
| TyG index, (IQR) | 8.82(8.42, 9.42) | 8.55(8.04, 8.91) | <0.001 |
| NLR, (IQR) | 6.93 (4.14, 11.17) | 5.28 (3.11, 8.09) | 0.001 |
| Fibrinogen, g/L (IQR) | 3.33 (2.76, 3.87) | 3.01 (2.62, 3.56) | 0.001 |
| Anterior circulation infarction, n (%) | 176 (67.69) | 128 (78.05) | 0.021 |
| Type of TOAST, n (%) |  |  | 0.428 |
| LAA | 178 (68.46) | 103 (62.80) |  |
| CE | 71 (27.31) | 51 (31.10) |  |
| Others | 11 (4.23) | 10 (6.10) |  |
| IVT, n (%) | 48 (18.46) | 33 (20.12) | 0.672 |
| Tirofiban treatment, n (%) | 89 (34.23) | 68 (41.46) | 0.133 |
| mTICI 2b~3, n (%) | 219 (84.23) | 155 (94.51) | 0.001 |
| END, n (%) | 63 (12.69) | 10 (6.10) | < 0.001 |
| sICH, n (%) | 59 (22.69) | 3 (1.83) | < 0.001 |

ASPECTS, Alberta Stroke Program Early Computed Tomography Score; BMI, Body Mass Index; CE, Cardio-embolism; DBP, Diastolic blood pressure; END, Early neurological deterioration; FBG, Fasting blood glucose; FTG, Fasting triglycerides; IVT, Intravenous thrombolysis; LAA, Large-artery atherosclerosis; mRS, modified Rankin Scale; mTICI, modified Thrombolysis in Cerebral Infarction; NIHSS, National Institutes of Health Stroke Scale; NLR, Neutrophil to lymphocyte ratio; SBP, Systolic blood pressure; sICH, symptomatic intracranial hemorrhage; TOAST, Trial of Org10172 in Acute Stroke Treatment; TyG index, Triglyceride-glucose index.

**Table S2.** Baseline and clinical characteristics of patients with END and without END.

|  | END (n=73) | No END (n=351) | *p* Value |
| --- | --- | --- | --- |
| Demographics |  |  |  |
| Age, years (IQR) | 65 (58, 75) | 65 (56, 71) | 0.361 |
| Gender, male, n (%) | 51 (69.86) | 247 (70.37) | 0.931 |
| BMI, kg/m^2^ (IQR) | 24.97 (23.24, 27.68) | 25.06 (23.44, 27.68) | 0.955 |
| Medical history |  |  |  |
| Pre-stroke, n (%) | 24 (32.88) | 96 (27.35) | 0.340 |
| Hypertension, n (%) | 53 (72.60) | 247 (70.37) | 0.703 |
| Diabetes mellitus, n (%) | 29 (39.73) | 90 (25.64) | 0.015 |
| Hyperlipidemia, n (%) | 28 (38.36) | 139 (39.60) | 0.843 |
| Coronary artery disease, n (%) | 27 (36.99) | 68 (19.37) | 0.001 |
| Atrial fibrillation, n (%) | 19 (26.03) | 79 (22.51) | 0.516 |
| Smoking, n (%) | 30 (41.10) | 147 (41.88) | 0.902 |
| Drinking, n (%) | 23 (31.51) | 122 (34.76) | 0.594 |
| NIHSS on admission, (IQR) | 14 (11, 20) | 16 (12, 21) | 0.021 |
| ASPECTS, (IQR) | 8 (7, 9) | 8 (7, 9) | 0.318 |
| blood pressure on admission, mmHg (IQR) |  |  |  |
| SBP | 157 (137, 170) | 150 (132, 166) | 0.109 |
| DBP | 86 (78, 97) | 83 (76, 92) | 0.232 |
| Laboratory examination |  |  |  |
| Blood glucose on admission, mmol/L (IQR) | 8.00 (6.45, 9.65) | 7.06 (6.10, 8.90) | 0.040 |
| FBG, mmol/L (IQR) | 7.94 (6.58, 11.27) | 6.93 (5.53, 8.96) | < 0.001 |
| FTG, mmol/L (IQR) | 1.00 (0.68, 1.57) | 1.04 (0.75, 1.46) | 0.828 |
| TyG index, (IQR) | 8.87 (8.46, 9.34) | 8.67 (8.27, 9.18) | 0.039 |
| NLR, (IQR) | 5.49 (3.99, 8.72) | 6.16 (3.65, 10.16) | 0.526 |
| Fibrinogen, g/L (IQR) | 3.21 (2.76, 3.89) | 3.17 (2.67, 3.68) | 0.349 |
| Anterior circulation infarction, n (%) | 44 (60.27) | 260 (74.07) | 0.017 |
| Type of TOAST, n (%) |  |  | 0.075 |
| LAA | 56 (76.71) | 225 (64.10) |  |
| CE | 13 (17.81) | 109 (31.05) |  |
| Others | 4 (5.48) | 17 (4.84) |  |
| IVT, n (%) | 21 (28.77) | 60 (17.09) | 0.021 |
| Tirofiban treatment, n (%) | 26 (35.62) | 131 (37.32) | 0.784 |
| mTICI 2b~3, n (%) | 57 (78.08) | 317 (90.31) | 0.003 |

ASPECTS, Alberta Stroke Program Early Computed Tomography Score; BMI, Body Mass Index; CE, Cardio-embolism; DBP, Diastolic blood pressure; END, Early neurological deterioration; FBG, Fasting blood glucose; FTG, Fasting triglycerides; IVT, Intravenous thrombolysis; LAA, Large-artery atherosclerosis; mRS, Modified Rankin Scale; mTICI, modified Thrombolysis in Cerebral Infarction; NIHSS, National Institutes of Health Stroke Scale; NLR, Neutrophil to lymphocyte ratio; SBP, Systolic blood pressure; TOAST, Trial of Org10172 in Acute Stroke Treatment; TyG index, Triglyceride-glucose index.

**Table S3.** Baseline and clinical characteristics of patients with sICH and without sICH.

|  | sICH (n=62) | No sICH (n=362) | *p* Value |
| --- | --- | --- | --- |
| Demographics |  |  |  |
| Age, years (IQR) | 69 (59, 76) | 64 (56, 71) | 0.008 |
| Gender, male, n (%) | 36 (58.06) | 262 (72.38) | 0.023 |
| BMI, kg/m^2^ (IQR) | 24.88 (23.02, 28.12) | 25.06 (23.44, 27.68) | 0.896 |
| Medical history |  |  |  |
| Pre-stroke, n (%) | 27 (43.55) | 93 (25.69) | 0.004 |
| Hypertension, n (%) | 47 (75.81) | 253 (69.89) | 0.344 |
| Diabetes mellitus, n (%) | 24 (38.71) | 95 (26.24) | < 0.001 |
| Hyperlipidemia, n (%) | 24 (38.71) | 143 (39.50) | 0.906 |
| Coronary artery disease, n (%) | 17 (27.42) | 78 (21.55) | 0.306 |
| Atrial fibrillation, n (%) | 20 (32.26) | 78 (21.55) | 0.065 |
| Smoking, n (%) | 19 (30.65) | 158 (43.65) | 0.055 |
| Drinking, n (%) | 14 (22.58) | 131 (36.19) | 0.037 |
| NIHSS on admission, (IQR) | 17 (12, 21) | 15 (12, 20) | 0.334 |
| ASPECTS, (IQR) | 8 (7, 9) | 8 (7, 9) | 0.745 |
| Blood pressure on admission, mmHg (IQR) |  |  |  |
| SBP | 158 (139, 175) | 150 (132, 166) | 0.022 |
| DBP | 86 (77, 94) | 83 (77, 93) | 0.915 |
| Laboratory examination |  |  |  |
| Blood glucose on admission, mmol/L (IQR) | 7.80 (6.50, 10.53) | 7.00 (6.10, 8.90) | 0.053 |
| FBG, mmol/L (IQR) | 8.27 (7.07, 13.27) | 6.92 (5.53, 8.93) | < 0.001 |
| FTG, mmol/L (IQR) | 1.01 (0.88, 1.63) | 1.02 (0.71, 1.43) | 0.094 |
| TyG index, (IQR) | 8.98 (8.52, 9.50) | 8.65 (8.26, 9.15) | < 0.001 |
| NLR, (IQR) | 6.91 (4.37, 10.34) | 5.89 (3.62, 9.86) | 0.095 |
| Fibrinogen, g/L (IQR) | 3.31 (2.74, 3.88) | 3.17 (2.68, 3.68) | 0.315 |
| Anterior circulation infarction, n (%) | 47 (75.81) | 257 (70.99) | 0.437 |
| Type of TOAST, n (%) |  |  | 0.440 |
| LAA | 41 (66.13) | 240 (66.30) |  |
| CE | 16 (25.81) | 106 (29.28) |  |
| Others | 5 (8.06) | 16 (4.42) |  |
| Intravenous thrombolysis, n (%) | 15 (24.29) | 66 (18.23) | 0.270 |
| Tirofiban treatment, n (%) | 15 (24.19) | 142 (39.23) | 0.024 |
| mTICI 2b~3, n (%) | 53 (85.48) | 321 (88.67) | 0.472 |

ASPECTS, Alberta Stroke Program Early Computed Tomography Score; BMI, Body Mass Index; CE, Cardio-embolism; DBP, Diastolic blood pressure; FBG, Fasting blood glucose; FTG, Fasting triglycerides; IVT, Intravenous thrombolysis; LAA, Large-artery atherosclerosis; mRS, Modified Rankin Scale; mTICI, modified Thrombolysis in Cerebral Infarction; NIHSS, National Institutes of Health Stroke Scale; NLR, Neutrophil to lymphocyte ratio; SBP, Systolic blood pressure; sICH, symptomatic intracranial hemorrhage; TOAST, Trial of Org10172 in Acute Stroke Treatment; TyG index, Triglyceride-glucose index.

**Table S4.** Baseline and clinical characteristics of patients with death and survival at 90 days.

|  | Dead(n=108) | Alive(n=316) | *p* Value |
| --- | --- | --- | --- |
| Demographics |  |  |  |
| Age, years (IQR) | 67 (58, 77) | 64 (56, 71) | 0.009 |
| Gender, male, n (%) | 69 (63,89) | 229 (72.47) | 0.092 |
| BMI, kg/m^2^ (IQR) | 25.42 (24.07, 28.69) | 24.84 (23.18, 27.68) | 0.119 |
| Medical history |  |  |  |
| Pre-stroke, n (%) | 39 (36.11) | 81 (25.63) | 0.037 |
| Hypertension, n (%) | 84 (77.78) | 216 (68.35) | 0.063 |
| Diabetes mellitus, n (%) | 42 (38.89) | 77 (24.37) | 0.004 |
| Hyperlipidemia, n (%) | 35 (32.41) | 132 (41.77) | 0.086 |
| Coronary artery disease, n (%) | 32 (29.63) | 63 (10.44) | 0.037 |
| Atrial fibrillation, n (%) | 29 (26.85) | 69 (21.84) | 0.286 |
| Smoking, n (%) | 36 (33.33) | 141 (44.62) | 0.040 |
| Drinking, n (%) | 25 (23.15) | 120 (37.97) | 0.005 |
| NIHSS on admission, (IQR) | 19 (14, 24) | 15 (11, 19) | < 0.001 |
| ASPECTS, (IQR) | 8 (7, 9) | 7 (7, 8) | < 0.001 |
| Blood pressure on admission, mmHg (IQR) |  |  |  |
| SBP | 155 (137, 170) | 149 (132, 166) | 0.022 |
| DBP | 86 (78, 93) | 83 (76, 93) | 0.513 |
| Laboratory examination |  |  |  |
| Blood glucose on admission, mmol/L (IQR) | 8.00 (6.60, 10.75) | 7.00 (6.10, 8.60) | < 0.001 |
| FBG, mmol/L (IQR) | 8.00 (6.60, 10.75) | 6.83 (5.44, 8.61) | < 0.001 |
| FTG, mmol/L (IQR) | 1.15 (0.85, 1.60) | 1.00 (0.71, 1.42) | 0.012 |
| TyG index, (IQR) | 8.93 (8.52, 9.47) | 8.60 (8.23, 9.07) | < 0.001 |
| NLR, (IQR) | 8.02 (4.89, 11.74) | 5.49 (3.10, 9.16) | < 0.001 |
| Fibrinogen, g/L (IQR) | 3.49 (2.78, 4.08) | 3.13 (2.67, 3.64) | 0.010 |
| Anterior circulation infarction, n (%) | 65 (60.19) | 239 (75.63) | 0.002 |
| Type of TOAST, n (%) |  |  | 0.072 |
| LAA | 80 (74.07) | 201 (63.61) |  |
| CE | 26 (24.07) | 96 (30.38) |  |
| Others | 2 (1.85) | 19 (6.01) |  |
| Intravenous thrombolysis, n (%) | 21 (19.44) | 60 (18.99) | 0.917 |
| Tirofiban treatment, n (%) | 36 (33.33) | 121 (38.29) | 0.357 |
| mTICI 2b~3, n (%) | 81 (75.00) | 293 (92.72) | < 0.001 |
| END, n (%) | 39 (36.11) | 34 (10.76) | < 0.001 |
| sICH, n (%) | 34 (31.48) | 28 (8.86) | < 0.001 |

ASPECTS, Alberta Stroke Program Early Computed Tomography Score; BMI, Body Mass Index; CE, Cardio-embolism; DBP, Diastolic blood pressure; END, Early neurological deterioration; FBG, Fasting blood glucose; FTG, Fasting triglycerides; IVT, Intravenous thrombolysis; LAA, Large-artery atherosclerosis; mRS, Modified Rankin Scale; mTICI, modified Thrombolysis in Cerebral Infarction; NIHSS, National Institutes of Health Stroke Scale; NLR, Neutrophil to lymphocyte ratio; SBP, Systolic blood pressure; sICH, symptomatic intracranial hemorrhage; TOAST, Trial of Org10172 in Acute Stroke Treatment; TyG index, Triglyceride-glucose index.

**Table S5.** Multivariate analysis of risk factors of poor outcome at 90 days.

|  | OR | 95% CI | *p* Value |
| --- | --- | --- | --- |
| Age | 1.010 | 0.987-1.032 | 0.403 |
| Female | 1.503 | 0.771-2.928 | 0.232 |
| BMI | 1.082 | 0.997-1.173 | 0.059 |
| Pre-stroke | 2.734 | 1.491-5.014 | 0.001 |
| Hypertension | 0.629 | 0.336-1.178 | 0.148 |
| Diabetes mellitus | 1.133 | 0.566-2.267 | 0.724 |
| Coronary artery disease | 1.690 | 0.854-3.346 | 0.132 |
| Drinking | 1.020 | 0.569-1.860 | 0.948 |
| NIHSS on admission | 1.048 | 1.011-1.087 | 0.012 |
| ASPECTS | 0.563 | 0.463-0.683 | < 0.001 |
| SBP | 1.009 | 0.998-1.021 | 0.118 |
| Blood glucose on admission | 1.037 | 0.931-1.157 | 0.507 |
| TyG index | 1.931 | 1.204-3.097 | 0.006 |
| NLR | 1.000 | 0.998-1.002 | 0.927 |
| Fibrinogen | 1.172 | 0.911-1.509 | 0.217 |
| posterior circulation infarction | 1.170 | 0.625-2.192 | 0.624 |
| mTICI 0~2a | 3.740 | 1.413-9.895 | 0.008 |
| END | 3.083 | 1.308-7.266 | 0.010 |
| sICH | 14.083 | 3.729-53.189 | < 0.001 |

ASPECTS, Alberta Stroke Program Early Computed Tomography Score; BMI, Body Mass Index; CI, Confidence interval; END, Early neurological deterioration; mTICI, modified Thrombolysis in Cerebral Infarction; NIHSS, National Institutes of Health Stroke Scale; NLR, Neutrophil to lymphocyte ratio; OR, Odds ratio; SBP, Systolic blood pressure; sICH, symptomatic intracranial hemorrhage; TyG index, Triglyceride-glucose index.

**Table S6.** Subgroup analysis of diabetes mellitus for association between TyG index and clinical outcomes.

| Diabetes mellitus | TyG index | Univariate analysis | | Multivariate analysis | |
| --- | --- | --- | --- | --- | --- |
|  |  | OR (95% CI) | *p* value | OR (95% CI) | *p* value |
| No |  |  |  |  |  |
| Poor outcome at 90 days^a^ | T1 | reference |  | reference |  |
|  | T2 | 1.555 (0.923, 2.622) | 0.097 | 1.343 (0.669, 2.698) | 0.407 |
|  | T3 | 2.276 (1.251, 4.140) | 0.007 | 0.948 (0.408, 2.200) | 0.901 |
| *p* for trend |  | 1.874 (1.198, 2.933) | 0.006 | 1.008 (0.539, 1.883) | 0.980 |
| END^b^ | T1 | reference |  | reference |  |
|  | T2 | 1.764 (0.813, 3.825) | 0.151 | 1.942 (0.854, 4.417) | 0.113 |
|  | T3 | 2,117 (0.935, 4.795) | 0.072 | 1,890 (0.774, 4.619) | 0.163 |
| *p* for trend |  | 1.734 (0.956, 3.144) | 0.070 | 1.599 (0.838, 3.051) | 0.154 |
| sICH^c^ | T1 | reference |  | reference |  |
|  | T2 | 1.995 (0.783, 5.081) | 0.148 | 1.835 (0.693, 4.858) | 0.221 |
|  | T3 | 4.950 (2.029, 12.075) | < 0.001 | 5.186 (2.034, 13.223) | 0.001 |
| *p* for trend |  | 3.334 (1.739, 6.391) | < 0.001 | 3.528 (1.764, 7.056) | < 0.001 |
| Mortality at 90 days^d^ | T1 | reference |  | reference |  |
|  | T2 | 3.067 (1.516, 6.203) | 0.002 | 3.488 (1.497, 8.126) | 0.004 |
|  | T3 | 4.023 (1.921, 8.425) | < 0.001 | 3.246 (1.311, 8.037) | 0.011 |
| *p* for trend |  | 2.669 (1.587, 4.488) | < 0.001 | 2.436 (1.268, 4.677) | 0.007 |
| Yes |  |  |  |  |  |
| Poor outcome at 90 days^a^ | T1 | reference |  | reference |  |
|  | T2 | 1.000 (0.256, 3.900) | 1.000 | 0.917 (0.154, 5.466) | 0.925 |
|  | T3 | 2.455 (0.627, 9.603) | 0.197 | 2.200 (0.368, 13.150) | 0.387 |
| *p* for trend |  | 2.368 (0.980, 5.719) | 0.055 | 2.275 (0.724, 7.153) | 0.160 |
| END^b^ | T1 | reference |  | reference |  |
|  | T2 | 1.200 (0.276, 5.209) | 0.808 | 1.187 (0.238, 5.933) | 0.835 |
|  | T3 | 0.824 (0.196, 3.456) | 0.791 | 0.882 (0.184, 4.225) | 0.876 |
| *p* for trend |  | 0.746 (0.309, 1.803) | 0.515 | 0.816 (0.297, 2.241) | 0.693 |
| sICH^c^ | T1 | reference |  | reference |  |
|  | T2 | 1.364 (0.252, 7.373) | 0.719 | 1.977 (0.316, 12.385) | 0.467 |
|  | T3 | 1.250 (0.244, 6.414) | 0.789 | 1.102 (0.182, 6.683) | 0.916 |
| *p* for trend |  | 1.040 (0.397, 2.722) | 0.937 | 0.758 (0.262, 2.193) | 0.609 |
| Mortality at 90 days^d^ | T1 | reference |  | reference |  |
|  | T2 | 1.345 (0.312, 5.798) | 0.691 | 2.106 (0.281, 15.796) | 0.469 |
|  | T3 | 2.000 (0.494, 8.092) | 0.331 | 2.849 (0.417, 19.456) | 0.285 |
| *p* for trend |  | 1.690 (0.733, 3.899) | 0.218 | 1.890 (0.584, 6.116) | 0.288 |

^a^Adjusted for significant covariates in Table S1. ^b^Adjusted for significant covariates in Table S2. ^c^Adjusted for significant covariates in Table S3. ^d^Adjusted for significant covariates in Table S4. CI, Confidence interval; END, Early neurological deterioration; OR, Odds ratio; sICH, symptomatic intracranial hemorrhage; TyG index, Triglyceride-glucose index.

**Table S7.** Subgroup analysis of NIHSS for association between TyG index and clinical outcomes.

| NIHSS | TyG index | Univariate analysis | | Multivariate analysis | |
| --- | --- | --- | --- | --- | --- |
|  |  | OR (95% CI) | *p* value | OR (95% CI) | *p* value |
| < 15 |  |  |  |  |  |
| Poor outcome at 90 days^a^ | T1 | reference |  | reference |  |
|  | T2 | 1.625 (0.803, 3.291) | 0.177 | 1.196 (0.422, 3.389) | 0.737 |
|  | T3 | 3.961 (1.862, 8.429) | < 0.001 | 2.272 (0.739, 6.985) | 0.152 |
| *p* for trend |  | 2.824 (1.602, 4.978) | < 0.001 | 1.891 (0.816, 4.385) | 0.138 |
| END^b^ | T1 | reference |  | reference |  |
|  | T2 | 3.424 (1.252, 9.364) | 0.017 | 3.539 (1.184, 10.575) | 0.024 |
|  | T3 | 4.709 (1.726, 12.847) | 0.002 | 4.359 (1.380, 13.765) | 0.012 |
| *p* for trend |  | 2.740 (1.411, 5.320) | 0.003 | 2.533 (1.160, 5.529) | 0.020 |
| sICH^c^ | T1 | reference |  | reference |  |
|  | T2 | 7.019 (1.503, 32.779) | 0.013 | 8.385 (1.686, 41.700) | 0.009 |
|  | T3 | 8.087 (1.725, 37.903) | 0.008 | 8.008 (1.548, 41.429) | 0.013 |
| *p* for trend |  | 2.992 (1.330, 6.729) | 0.008 | 2.746 (1.127, 6.590) | 0.026 |
| Mortality at 90 days^d^ | T1 | reference |  | reference |  |
|  | T2 | 3.067 (1.516, 6.203) | 0.002 | 5.111 (0.813, 32.148) | 0.082 |
|  | T3 | 4.023 (1.921, 8.425) | < 0.001 | 3.864 (0.663, 22.518) | 0.133 |
| *p* for trend |  | 2.708 (1.274, 5.758) | 0.010 | 2.137 (0.645, 7.077) | 0.214 |
| ≥ 15 |  |  |  |  |  |
| Poor outcome at 90 days^a^ | T1 | reference |  | reference |  |
|  | T2 | 1.670 (0.859, 3.247) | 0.130 | 1.115 (0.469, 2.649) | 0.805 |
|  | T3 | 2.497 (1.236, 5.045) | 0.011 | 0.772 (0.284, 2.096) | 0.611 |
| *p* for trend |  | 1.995 (1.170, 3.399) | 0.011 | 0.827 (0.389, 1.759) | 0.621 |
| END^b^ | T1 | reference |  | reference |  |
|  | T2 | 1.182 (0.479, 2.921) | 0.717 | 0.924 (0.348, 2.458) | 0.875 |
|  | T3 | 0.849 (0.325, 2.219) | 0.739 | 0.564 (0.185, 1.714) | 0.312 |
| *p* for trend |  | 0.875 (0.435, 1.760) | 0.708 | 0.645 (0.282, 1.472) | 0.298 |
| sICH^c^ | T1 | reference |  | reference |  |
|  | T2 | 1.093 (0.399, 2.997) | 0.862 | 0.986 (0.334, 2.912) | 0.979 |
|  | T3 | 2.686 (1.098, 6.575) | 0.030 | 2.736 (0.973, 7.698) | 0.056 |
| *p* for trend |  | 2.273 (1.157, 4.467) | 0.017 | 2.344 (1.068, 5.145) | 0.034 |
| Mortality at 90 days^d^ | T1 | reference |  | reference |  |
|  | T2 | 1.345 (0.312, 5.798) | 0.691 | 2.769 (1.139, 6.731) | 0.025 |
|  | T3 | 2.000 (0.494, 8.092) | 0.331 | 3.199 (1.282, 7.983) | 0.013 |
| *p* for trend |  | 2.604 (1.547, 4.382) | < 0.001 | 2.167 (1.138, 4.127) | 0.019 |

^a^Adjusted for significant covariates in Table S1. ^b^Adjusted for significant covariates in Table S2. ^c^Adjusted for significant covariates in Table S3. ^d^Adjusted for significant covariates in Table S4. CI, Confidence interval; END, Early neurological deterioration; NIHSS, National Institutes of Health Stroke Scale; OR, Odds ratio; sICH, symptomatic intracranial hemorrhage; TyG index, Triglyceride-glucose index.

**Table S8.** Subgroup analysis of TOAST for association between TyG index and clinical outcomes.

| TOAST | TyG index | Univariate analysis | | Multivariate analysis | |
| --- | --- | --- | --- | --- | --- |
|  |  | OR (95% CI) | *p* value | OR (95% CI) | *p* value |
| LAA |  |  |  |  |  |
| Poor outcome at 90 days | T1 | reference |  | reference |  |
|  | T2 | 1.354 (0.742, 2.472) | 0.324 | 0.699 (0.308, 1.587) | 0.392 |
|  | T3 | 2.501 (1.359, 4.601) | 0.003 | 0.736 (0.304, 1.783) | 0.497 |
| *p* for trend |  | 2.005 (1.272, 3.161) | 0.003 | 0.811 (0.418, 1.571) | 0.534 |
| END | T1 | reference |  | reference |  |
|  | T2 | 1.775 (0.812, 3.881) | 0.151 | 1.483 (0.634, 3.468) | 0.363 |
|  | T3 | 1.620 (0.753, 3.485) | 0.217 | 1.279 (0.525, 3.118) | 0.588 |
| *p* for trend |  | 1.337 (0.781, 2.288) | 0.290 | 1.135 (0.598, 2.156) | 0.698 |
| sICH | T1 | reference |  | reference |  |
|  | T2 | 3.591 (1.132, 11.394) | 0.030 | 4.938 (1.408, 17.311) | 0.013 |
|  | T3 | 5.408 (1.791, 16.332) | 0.003 | 6.345 (1.877, 21.445) | 0.003 |
| *p* for trend |  | 2.811 (1.452, 5.442) | 0.002 | 2.894 (1.380, 6.071) | 0.005 |
| Mortality at 90 days | T1 | reference |  | reference |  |
|  | T2 | 2.496 (1.168, 5.334) | 0.018 | 2.141 (0.849, 5.397) | 0.107 |
|  | T3 | 3.676 (1.780, 7.592) | < 0.001 | 2.240 (0.892, 5.625) | 0.086 |
| *p* for trend |  | 2.414 (1.468, 3.968) | 0.001 | 1.729 (0.901, 3.319) | 0.100 |
| CE |  |  |  |  |  |
| Poor outcome at 90 days | T1 | reference |  | reference |  |
|  | T2 | 2.193 (0.959, 5.014) | 0.063 | 1.630 (0.470, 5.653) | 0.441 |
|  | T3 | 3.857 (1.380, 10.783) | 0.010 | 2.549 (0.459, 14.146) | 0.284 |
| *p* for trend |  | 2.860 (1.320, 6.195) | 0.008 | 2.046 (0.571, 7.331) | 0.272 |
| END | T1 | reference |  | reference |  |
|  | T2 | 2.250 (0.528, 9.591) | 0.273 | 2.352 (0.493, 11.219) | 0.283 |
|  | T3 | 2.500 (0.517, 12.099) | 0.255 | 1.973 (0.314, 12.399) | 0.469 |
| *p* for trend |  | 1.890 (0.630, 5.669) | 0.256 | 1.558 (0.430, 5.642) | 0.500 |
| sICH | T1 | reference |  | reference |  |
|  | T2 | 2.250 (0.528, 9.591) | 0.273 | 1.422 (0.294, 6.869) | 0.662 |
|  | T3 | 5.000 (1.175, 21.279) | 0.029 | 2.709 (0.519, 14.136) | 0.237 |
| *p* for trend |  | 3.265 (1.163, 9.167) | 0.025 | 2.159 (0.640, 7.287) | 0.215 |
| Mortality at 90 days | T1 | reference |  | reference |  |
|  | T2 | 4.333 (1.295, 14.505) | 0.017 | 5.117 (1.101, 23.789) | 0.037 |
|  | T3 | 5.211 (1.427, 19.021) | 0.012 | 4.161 (0.788, 21.973) | 0.093 |
| *p* for trend |  | 2.972 (1.263, 6.996) | 0.013 | 2.396 (0.776, 7.398) | 0.129 |

^a^Adjusted for significant covariates in Table S1. ^b^Adjusted for significant covariates in Table S2. ^c^Adjusted for significant covariates in Table S3. ^d^Adjusted for significant covariates in Table S4. The sample size of “other” type was insufficient for statistical analysis. CE, Cardio-embolism; CI, Confidence interval; END, Early neurological deterioration; LAA, Large-artery atherosclerosis; OR, Odds ratio; sICH, symptomatic intracranial hemorrhage; TOAST, Trial of Org10172 in Acute Stroke Treatment; TyG index, Triglyceride-glucose index.

**Table S9.** Subgroup analysis for association between TyG index (per unit) and clinical outcomes.

| Subgroup | clinical outcomes | Univariate analysis | | Multivariate analysis | |
| --- | --- | --- | --- | --- | --- |
|  |  | OR (95% CI) | *p* value | OR (95% CI) | *p* value |
| Diabetes mellitus |  |  |  |  |  |
| No | Poor outcome at 90 days^a^ | 2.407 (1.584, 3.656) | < 0.001 | 1.530 (0.878, 2.665) | 0.134 |
|  | END^b^ | 1.614 (0.959, 2.716) | 0.072 | 1.580 (0.881, 2.835) | 0.125 |
|  | sICH^c^ | 3.229 (1.800, 5.792) | < 0.001 | 3.594 (1.922, 6.718) | < 0.001 |
|  | Mortality at 90 days^d^ | 2.605 (1.616, 4.198) | < 0.001 | 2.013 (1.106, 3.663) | 0.022 |
| Yes | Poor outcome at 90 days^a^ | 3.120 (1.449, 6.717) | 0.004 | 4.187 (1.417, 12.371) | 0.010 |
|  | END^b^ | 0.773 (0.406, 1.471) | 0.432 | 0.793 (0.374, 1.679) | 0.544 |
|  | sICH^c^ | 1.112 (0.571, 2.165) | 0.755 | 0.954 (0.435, 2.093) | 0.907 |
|  | Mortality at 90 days^d^ | 1.483 (0.841, 2.612) | 0.173 | 1.302 (0.570, 2.975) | 0.532 |
| NIHSS |  |  |  |  |  |
| < 15 | Poor outcome at 90 days^a^ | 3.347 (1.938, 5.782) | < 0.001 | 2.940 (1.263, 6.843) | 0.012 |
|  | END^b^ | 2.464 (1.380, 4.400) | 0.002 | 2.550 (1.228, 5.292) | 0.012 |
|  | sICH^c^ | 2.564 (1.296, 5.074) | 0.007 | 2.441 (1.075, 5.541) | 0.033 |
|  | Mortality at 90 days^d^ | 2.436 (1.278, 4.644) | 0.007 | 1.897 (0.665, 5.407) | 0.231 |
| ≥ 15 | Poor outcome at 90 days^a^ | 2.557 (1.609, 4.062) | < 0.001 | 1.448 (0.788, 2.661) | 0.233 |
|  | END^b^ | 0.943 (0.558, 1.595) | 0.827 | 0.695 (0.366, 1.322) | 0.268 |
|  | sICH^c^ | 1.950 (1.205, 3.157) | 0.007 | 2.557 (1.340, 4.877) | 0.004 |
|  | Mortality at 90 days^d^ | 2.087 (1.400, 3.111) | < 0.001 | 1.611 (0.971, 2.673) | 0.065 |
| TOAST |  |  |  |  |  |
| LAA | Poor outcome at 90 days^a^ | 2.527 (1.680, 3.803) | < 0.001 | 1.375 (0.782, 2.420) | 0.269 |
|  | END^b^ | 1.255 (0.830, 1.899) | 0.281 | 1.135 (0.671, 1.921) | 0.637 |
|  | sICH^c^ | 2.141 (1.336, 3.431) | 0.002 | 2.202 (1.242, 3.907) | 0.007 |
|  | Mortality at 90 days^d^ | 2.104 (1.426, 3.106) | < 0.001 | 1.357 (0.809, 2.276) | 0.247 |
| CE | Poor outcome at 90 days^a^ | 3.553 (1.758, 7.178) | < 0.001 | 3.282 (1.055,10.207) | 0.040 |
|  | END^b^ | 1.406 (0.569, 3.478) | 0.460 | 1.134 (0.383, 3.356) | 0.821 |
|  | sICH^c^ | 3.190 (1.335, 7.620) | 0.009 | 2.720 (0.900, 8.217) | 0.076 |
|  | Mortality at 90 days^d^ | 2.347 (1.148, 4.801) | 0.019 | 2.073 (0.764, 5.625) | 0.152 |

^a^Adjusted for significant covariates in Table S1. ^b^Adjusted for significant covariates in Table S2. ^c^Adjusted for significant covariates in Table S3. ^d^Adjusted for significant covariates in Table S4. The sample size of “other” type was insufficient for statistical analysis. CE, Cardio-embolism; CI, Confidence interval; END, Early neurological deterioration; LAA, Large-artery atherosclerosis; NIHSS, National Institutes of Health Stroke Scale; OR, Odds ratio; sICH, symptomatic intracranial hemorrhage; TOAST, Trial of Org10172 in Acute Stroke Treatment; TyG index, Triglyceride-glucose index.

**Table S10.** Subgroup analysis for the relationship between the TyG index and clinical outcomes after adjustment.

| Subgroup | clinical outcomes | RCS TyG index | RCS (TyG index)' | *p* for non-linearity |
| --- | --- | --- | --- | --- |
| Diabetes mellitus |  |  |  |  |
| No | Poor outcome at 90 days^a^ | 3.545 (1.114, 11.279) | 0.281 (0.064, 1.237) | 0.090 |
|  | END^b^ | 4.482 (0.799, 25.128) | 0272 (0.038, 1.942) | 0.170 |
|  | sICH^c^ | 8.571 (0.832, 88.347) | 0.406 (0.042, 3.946) | 0.420 |
|  | Mortality at 90 days^d^ | 8.249 (1.148, 59.285) | 0.199 (0.026, 1.543) | 0.103 |
| Yes | Poor outcome at 90 days^a^ | 0.156 (0.010, 2.498) | 1135.674 (3.390, 380490.316) | 0.004 |
|  | END^b^ | 1.368 (0.235, 7.970) | 0.446 (0.042, 4.749) | 0.450 |
|  | sICH^c^ | 0.533 (0.088, 3.217) | 2.242 (0.238, 21.154) | 0.484 |
|  | Mortality at 90 days^d^ | 3.095 (0.418, 22.937) | 0.313 (0.028, 3.561) | 0.344 |
| NIHSS |  |  |  |  |
| < 15 | Poor outcome at 90 days^a^ | 1.367 (0.238, 7.859) | 3.347 (0284, 39.488) | 0.332 |
|  | END^b^ | 6.234 (0.838, 46.367) | 0.331 (0.035, 3.115) | 0.319 |
|  | sICH^c^ | 60.517 (2.187, 1674.876) | 0.025 (0.001, 0.772) | 0.015 |
|  | Mortality at 90 days^d^ | 28.217 (0.747, 1066.274) | 0.035 (0.001, 2.143) | 0.814 |
| ≥ 15 | Poor outcome at 90 days^a^ | 1.355 (0.467, 3.932) | 1.126 (0.237, 5.336) | 0.881 |
|  | END^b^ | 1.645 (0.368, 7.359) | 0.280 (0.040, 1.947) | 0.171 |
|  | sICH^c^ | 2.085 (0.375, 11.590) | 1.274 (0.191, 8.519) | 0.804 |
|  | Mortality at 90 days^d^ | 4.016 (0.969, 16.638) | 0.325 (0.067, 1.579) | 0.149 |
| TOAST |  |  |  |  |
| LAA | Poor outcome at 90 days^a^ | 0.635 (0.223, 1.807) | 4.130 (0.772, 22.088) | 0.086 |
|  | END^b^ | 1.709 (0.485, 6.026) | 0.559 (0.113, 2.769) | 0.466 |
|  | sICH^c^ | 10.613 (1.346, 83.686) | 0.153 (0.015, 1.522) | 0.090 |
|  | Mortality at 90 days^d^ | 2.337 (0.561, 9.730) | 0.497 (0.093, 2.662) | 0.404 |
| CE | Poor outcome at 90 days^a^ | 5.805 (0.553, 60.924) | 0.444 (0.025, 7.808) | 0.581 |
|  | END^b^ | 20.281 (0.508, 809.887) | 0.025 (0.000, 1.788) | 0.044 |
|  | sICH^c^ | 5.185 (0.184, 145.885) | 0.492 (0.017, 14.435) | 0.674 |
|  | Mortality at 90 days^d^ | 28.837 (0.857, 969.788) | 0.046 (0.001, 1.874) | 0.069 |

^a^Adjusted for significant covariates in Table S1. ^b^Adjusted for significant covariates in Table S2. ^c^Adjusted for significant covariates in Table S3. ^d^Adjusted for significant covariates in Table S4. The sample size of “other” type was insufficient for statistical analysis. CE, Cardio-embolism; END, Early neurological deterioration; LAA, Large-artery atherosclerosis; NIHSS, National Institutes of Health Stroke Scale; RCS, Restricted cubic splines; sICH, symptomatic intracranial hemorrhage; TOAST, Trial of Org10172 in Acute Stroke Treatment; TyG index, Triglyceride-glucose index.

**Table S11.** Baseline characteristics between included and excluded patients.

|  | Included patients (n=424) | Excluded patients (n=131^#^) | *p* Value |
| --- | --- | --- | --- |
| Demographics |  |  |  |
| Age, years (IQR) | 65 (56, 72) | 65 (59, 75) | 0.176 |
| Gender, male, n (%) | 298 (73.76) | 89 (67.94) | 0.610 |
| BMI, kg/m^2^ (IQR) | 25.02 (23.44, 27.68) | 24.49 (22.04, 26.67) | 0.014 |
| Medical history |  |  |  |
| Pre-stroke, n (%) | 120 (28.30) | 40 (30.53) | 0.622 |
| Hypertension, n (%) | 300 (70.75) | 80 (61.07) | 0.037 |
| Diabetes mellitus, n (%) | 119 (28.07) | 31 (23.66) | 0.321 |
| Hyperlipidemia, n (%) | 167 (39.39) | 28 (21.37) | < 0.001 |
| Coronary artery disease, n (%) | 95 (22.41) | 25 (19.08) | 0.420 |
| Atrial fibrillation, n (%) | 98 (23.11) | 36 (27.48) | 0.307 |
| Smoking, n (%) | 177 (41.75) | 46 (35.11) | 0.176 |
| Drinking, n (%) | 145 (34.20) | 42 (32.06) | 0.651 |
| NIHSS on admission, (IQR) | 16 (12, 20) | 17 (12, 20) | 0.547 |
| ASPECTS, (IQR) | 8 (7, 9) | 8 (7, 9) | 0.833 |
| Blood pressure on admission, mmHg (IQR) |  |  |  |
| SBP | 150 (133, 167) | 150 (139, 166) | 0.534 |
| DBP | 84 (77, 93) | 82 (72, 90) | 0.345 |
| Anterior circulation infarction, n (%) | 304 (71.70) | 101 (77.10) | 0.224 |
| Type of TOAST, n (%) |  |  | 0.587 |
| LAA | 281 (66.27) | 93 (70.99) |  |
| CE | 122 (28.77) | 33 (25.19) |  |
| Others | 21 (4.95) | 5 (3.82) |  |
| IVT, n (%) | 81 (19.10) | 29 (22.14) | 0.446 |
| Tirofiban treatment, n (%) | 157 (37.03) | 38 (29.01) | 0.093 |
| mTICI 2b~3, n (%) | 374 (88.21) | 121 (92.37) | 0.180 |

^#^131 cases with complete baseline data of 170 excluded patients were analyzed. ASPECTS, Alberta Stroke Program Early Computed Tomography Score; BMI, Body Mass Index; CE, Cardio-embolism; DBP, Diastolic blood pressure; IVT, Intravenous thrombolysis; LAA, Large-artery atherosclerosis; mRS, modified Rankin Scale; mTICI, modified Thrombolysis in Cerebral Infarction; NIHSS, National Institutes of Health Stroke Scale; SBP, Systolic blood pressure; TOAST, Trial of Org10172 in Acute Stroke Treatment.

**
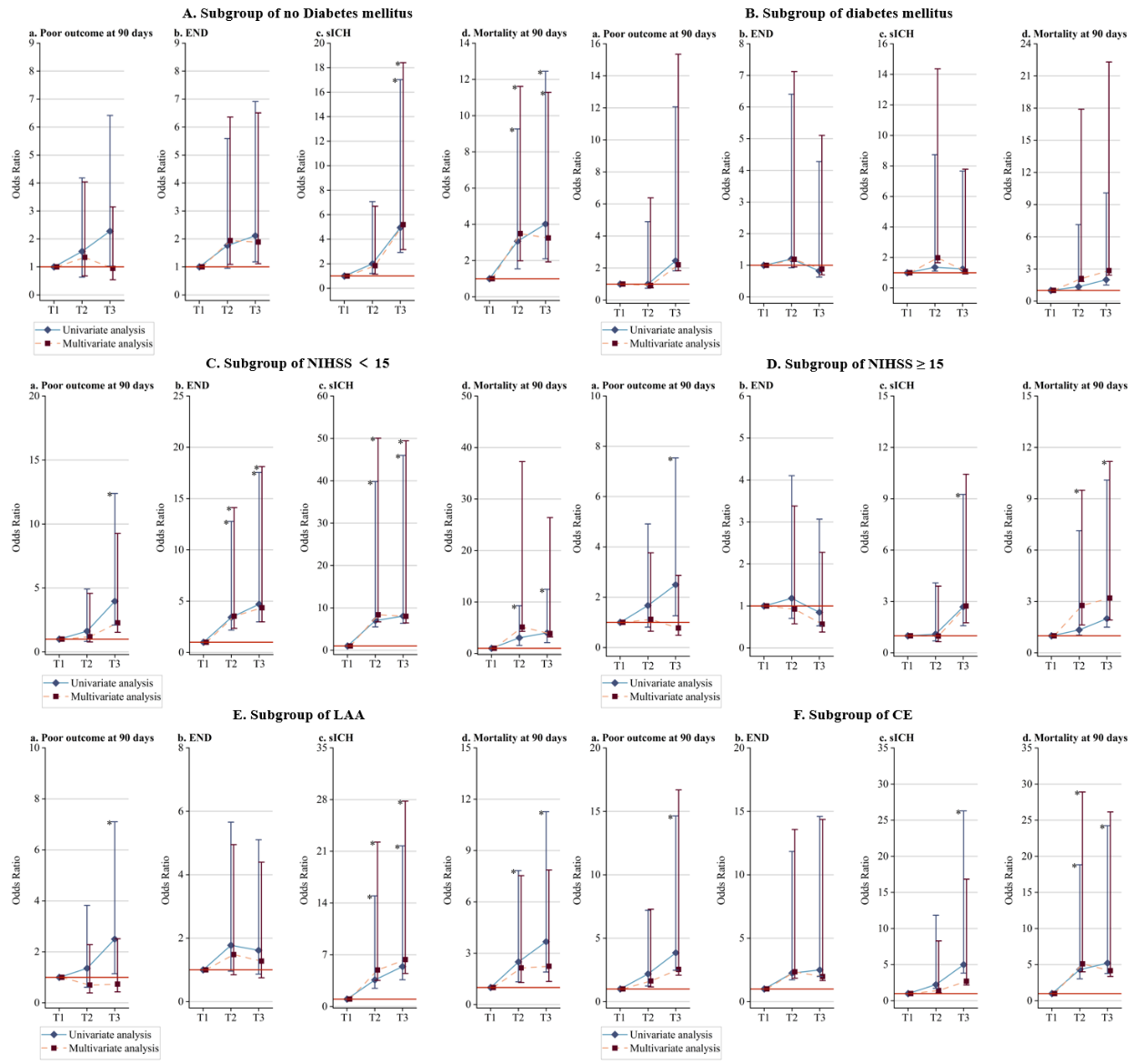
Figure S1**. Subgroup analysis for the clinical outcomes measures by tertiles of TyG index. *Statistical significance (*p* < 0.05). Adjusted for significant covariates in Table S1-S4. The sample size of “other” type was insufficient for statistical analysis.CE, Cardio-embolism; END, Early neurological deterioration; LAA, Large-artery atherosclerosis; NIHSS, National Institutes of Health Stroke Scale; sICH, symptomatic intracranial hemorrhage.


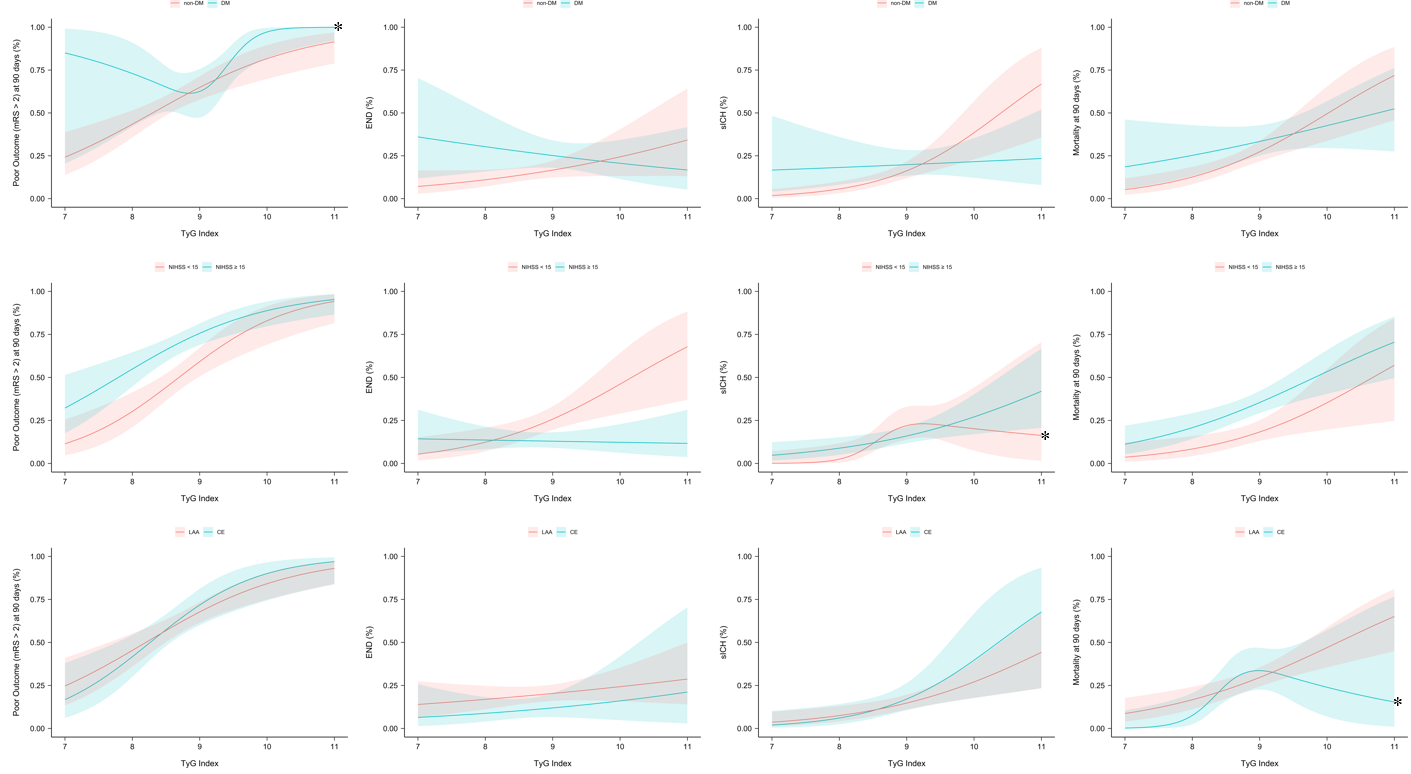


**Figure S2**. Subgroup analysis for the relationship between the TyG index and clinical outcomes. *Statistical significance (*p* for non-linearity < 0.05). The sample size of “other” type was insufficient for statistical analysis. CE, Cardio-embolism; DM, Diabetes mellitus; END, Early neurological deterioration; LAA, Large-artery atherosclerosis; NIHSS, National Institutes of Health Stroke Scale; sICH, symptomatic intracranial hemorrhage.
